# An Intervention to Increase Advance Care Planning Among Older Adults With Advanced Cancer: A Randomized Clinical Trial

**DOI:** 10.1001/jamanetworkopen.2025.9150

**Published:** 2025-05-09

**Authors:** Angelo E. Volandes, Yuchiao Chang, Joshua R. Lakin, Michael K. Paasche-Orlow, Charlotta Lindvall, Seth N. Zupanc, Diana Martins-Welch, Maria T. Carney, Edith A. Burns, Jennifer Itty, Kaitlin Emmert-Tangredi, Narda J. Martin, Shreya Sanghani, Jon Tilburt, Kathryn I. Pollak, Aretha Delight Davis, Cynthia Garde, Michael J. Barry, Areej El-Jawahri, Lisa Quintiliani, Kate Sciacca, Julie Goldman, James A. Tulsky

**Affiliations:** 1 Department of Medicine, Dartmouth Health, Lebanon, New Hampshire; 2 Geisel School of Medicine, Hanover, New Hampshire; 3 Department of Medicine, Massachusetts General Hospital, Boston; 4 ACP Decisions, Waban, Massachusetts; 5 Department of Supportive Oncology, Dana-Farber Cancer Institute, Boston, Massachusetts; 6 Department of Medicine, Brigham and Women’s Hospital, Boston, Massachusetts; 7 Harvard Medical School, Boston, Massachusetts; 8 Department of Medicine, Tufts University School of Medicine, Tufts Medical Center, Boston, Massachusetts; 9 School of Medicine, University of California San Francisco, San Francisco; 10 Institute of Health System Science, Feinstein Institutes for Medical Research, Manhasset, New York; 11 Department of Medicine, Zucker School of Medicine Hofstra/Northwell, New Hyde Park, New York; 12 Department of Medicine, Mayo Clinic, Rochester, Minnesota; 13 Department of Population Health Sciences, Duke University School of Medicine and Duke Cancer Institute, Durham, North Carolina

## Abstract

**Importance:**

Many older adults with advanced cancer never communicate goals of care or treatment preferences to their clinicians, raising the risk that care received will not match their values. Scalable models of care may help surmount this barrier.

**Objective:**

To test whether a combined patient and clinician intervention increased the rate of advance care planning (ACP) documentation in large health care systems.

**Design, Setting, and Participants:**

This stepped-wedge cluster randomized clinical trial using an open cohort design included patients aged 65 years or older with advanced cancer seen at oncology clinics in 3 health care systems located in the US South, Midwest, and Mid-Atlantic regions from April 1, 2020, to November 30, 2022. Data collection ended in 2024.

**Intervention:**

The intervention involved delivering brief evidence-based patient-facing video decision aids available in 25 languages as well as goals-of-care communication training to oncology clinicians. Patients in the control period received usual care.

**Main Outcomes and Measures:**

The primary outcome was ACP documentation, which included any electronic health record documentation of a goals-of-care conversation, palliative care, hospice, or limitation of life-sustaining treatments, identified via a validated natural language processing program. Analysis was performed on an intention-to-treat basis.

**Results:**

Twenty-nine practices, comprising 13 800 unique eligible patients with a total of 29 357 repeated measurements, were included (mean [SD] age, 74.5 [6.6] years; 52.3% men [15 344 of 29 357 measurements]). The proportion of patients with ACP documentation was greater in the intervention phase compared with the usual care phase (adjusted rate difference, 6.8% [95% CI, 2.8%-10.8%]; *P* < .001). ACP documentation in the intervention phase occurred among 3980 of 15 754 patients (25.3%) (goals-of-care conversation, 21.4% [3377 of 15 754]; palliative care, 9.6% [1517 of 15 754]; hospice, 5.4% [847 of 15 754]; and limitation of life-sustaining treatments, 7.2% [1128 of 15 754]). In comparison, ACP documentation in the usual care phase occurred among 2834 of 13 603 patients (20.8%) (goals-of-care conversation, 16.8% [2281 of 13 603]; palliative care, 9.5% [1287 of 13 603]; hospice, 5.3% [724 of 13 603]; and limitation of life-sustaining treatments, 8.4% [1149 of 13 603]).

**Conclusions and Relevance:**

In this stepped-wedge cluster randomized clinical trial for older adults with advanced cancer, a bundled evidence-based decision aid and communication training intervention increased the proportion of older patients with ACP documentation. This approach offers an innovative paradigm with a clinically meaningful increase in ACP documentation, a widely used quality metric that reflects high-quality patient-centered care delivery.

**Trial Registration:**

ClinicalTrials.gov Identifier: NCT03609177

## Key Points

**Question:**

Can use of the combination of evidence-based patient video decision aids with clinician communication skills training in ambulatory oncology practices improve advance care planning (ACP) documentation among older patients (aged ≥65 years) with advanced cancer?

**Findings:**

In this randomized clinical trial that included 13 800 unique patients at 29 clinics in 3 health care systems, patients randomized to clinics receiving the bundled intervention had significantly more ACP documentation than patients randomized to clinics receiving usual care (25.3% vs 20.8%).

**Meaning:**

The results of this study suggest that implementing patient video decision aids along with bolstering clinician communication skills can improve clinical ACP documentation for older adults with advanced cancer.

## Teaser

This stepped-wedge cluster randomized clinical trial assesses whether a combined patient and clinician intervention increased the rate of advance care planning documentation among older patients with advanced cancer in large health care systems.

## Introduction

Discussing goals of care between patients and clinicians has been reported to promote patient-centered high-quality care.^1,2^ Eliciting patient preferences for current and future medical care empowers them in the advanced stages of illness to align intervention intensity with their goals.^3^ Despite accumulating evidence of the benefits of these discussions, documentation of advance care planning (ACP) activity in the electronic health record (EHR) remains low and inconsistent for most health care systems, increasing the risk that patients will not receive care that matches their goals.^4,5^

Barriers to such discussions include both limited patient awareness and hesitancy to broach emotionally fraught topics.^6,7^ Furthermore, without clear guiding conversation frameworks, clinicians lack confidence initiating these discussions.^8,9^ Video decision aids can help inform patients of potential choices^10,11,12,13^ and communication training can equip clinicians to engage in tough conversations.^8,9^ The emergency of the COVID-19 pandemic highlighted the urgent need for health care systems to adopt scalable interventions to improve ACP communication and documentation, especially for older patients (aged ≥65 years) with serious illnesses such as advanced cancer.^14^

We conducted a large pragmatic randomized clinical trial testing the effect of a combined patient-facing video decision aid platform with advanced clinician communication skills training on ACP documentation for older patients with advanced cancer compared with usual care. We hypothesized that our bundled intervention delivered proactively in the ambulatory setting would increase the rate of ACP documentation for older adults with advanced cancer.

## Methods

### Trial Design and Oversight

We conducted a large, pragmatic multicenter stepped-wedge cluster randomized clinical trial comparing a combined video decision aid and communication skills training program (intervention) with usual care (control). The trial protocol is available in Supplement 1. Because the intervention included training clinicians and using decision aids in ambulatory clinics, randomization was performed at the ambulatory unit level.^15,16^ This design was chosen to facilitate implementation while minimizing the risk of contamination during the control period.^17^ The trial was conducted between April 1, 2020, and November 30, 2022. Data collection ended in 2024. A data safety and monitoring board was established and convened. Institutional review board approval was secured through Dana-Farber Cancer Institute. Given the minimal risk nature of the intervention testing an enhanced standard of care against usual care, the institutional review board granted a waiver of individual informed consent. Nonetheless, the study team used broadcast notification techniques (eg, flyers in clinic offices) to notify patients of the ongoing trial.^18^ This study followed the Consolidated Standards of Reporting Trials (CONSORT) reporting guideline.

As detailed in the article on the study protocol,^19^ the study initially began with 36 clinics, with a group of 6 clinics exposed to the intervention 6 months before COVID-19 (September 2019). In March 2020, all trial activities ceased. After initially randomizing the 36 clinics in 2019, we rerandomized the remaining 30 clinics in April 2020 to account for the temporal effects related to the pandemic. In this study, we report data from 29 of the 30 clinics, as 1 clinic disaffiliated with its health system and data access was lost.

Each clinic (cluster) began in the control phase and transitioned to the intervention phase at a randomly assigned time (wedge) over 2.5 years. Twenty-nine disease-based oncology clinics were included from 3 health care systems located in geographically diverse areas: the South, the Midwest, and the Mid-Atlantic region. For the purpose of reporting results, the 3 health systems preferred to remain anonymous. The geographic locations described are not necessarily the A, B, and C labels used in this study. The order of clusters for intervention initiation was randomly determined (eFigure in Supplement 2). After a 6-month baseline run-in period, new clinics were randomly introduced to the intervention every 6 months over the course of 4 steps (step 1: 6 clinics; step 2: 9 clinics; step 3: 9 clinics; step 4: 5 clinics). Once clinics were exposed to the intervention, they remained exposed for the remainder of the study.

### Patients

Eligible patients (1) were aged 65 years or older at the start of each step, (2) were seen by any clinician in one of the participating clinics, and (3) had advanced cancer. To be included, patients also had to have a minimum of 2 prior visits (in person or telehealth) to the clinic, and at least 1 visit during the 6 months of the step.

Advanced cancer for this population was defined as follows: all patients seen for lymphoma, leukemia, myeloma, or brain tumor; for all other solid tumor malignant neoplasms, we used *International Statistical Classification of Diseases and Related Health Problems, Tenth Revision* codes (and all subcodes) for C77.X, C78.X, and C79.X (C77: secondary and unspecified malignant neoplasm of lymph nodes; C78: secondary malignant neoplasm of respiratory and digestive organs; C79: secondary malignant neoplasm of other and unspecified sites).^20^ There were no exclusion criteria.^21^ Clinical data from patients who had affirmatively declined to have their record used for research and all patients with an EU address were excluded from the final analysis.

### Intervention

The intervention bundled 2 previously tested individual interventions. We hypothesized that when combined the bundle would improve goals-of-care communication in an additive manner. The patient-facing certified^22^ video decision aids addressing a range of topics (eg, goals of care, palliative care, hospice, and limitation of life-sustaining interventions) are grounded in the theory of shared decision-making^23,24^ and are designed to assist patients and decision-makers with envisioning their goals of care and to facilitate communication.^10,11,13^ They are available in 25 languages and have been shown to improve ACP knowledge and certainty,^10,13,25^ ACP documentation,^26^ and goal-concordant care.^25^

A clinician communication skills training program (VitalTalk) has been disseminated to more than 50 000 clinicians internationally and is the core serious illness training model used in 6 of the 10 Alliance of Dedicated Cancer Centers hospitals.^27,28,29^ This training program is based on learning a framework for conducting these conversations, followed by practice and feedback on one’s own communication skills. This framework increases the ability of clinicians to deliver serious news clearly, respond to emotion, elicit patients’ goals, and increase trust in the oncologist.

As each clinic was introduced into the intervention phase, its clinicians (physicians and advanced practice clinicians) became eligible to receive a half-day live virtual communication training. Fidelity of the communication training was assessed as the proportion of eligible staff trained over a 6-month period. All patients with advanced cancer affiliated with the clinic had the opportunity to use a video decision aid remotely or in person as directed by their clinician. Fidelity of the video component was monitored by tracking video use.

### Outcomes and Data Collection

The primary end point was ACP documentation in the EHR during a clinician encounter. We used a validated natural language processing (NLP) software (ClinicalRegex, version 1.1.2; Lindvall Lab, Dana-Farber Cancer Institute) for NLP-assisted human adjudication of ACP communication documented in the free text of clinical notes as our primary outcome, as previously reported.^19,30,31,32^ ACP communication included any documentation of a conversation about goals of care, limitation of life-sustaining treatment, palliative care, or hospice (eTable in Supplement 2). Research staff were blinded to treatment group assignments and adjudicated within the software. Specifically, text blocks identified by NLP from all oncology EHR notes for each patient were presented in the software for evaluation by trained research staff at each site. Training datasets were used to conduct ongoing quality assurance and to ensure consistent adjudication at each site.

### Statistical Analysis

Our primary analytical approach used an intention-to-treat analysis, with no special allowance for noncompliance or nonadherence. The outcomes during the intervention periods were compared with outcomes during the control periods. With the open cohort design, individuals may leave and others may join during the study. In addition, a single patient could contribute data to more than 1 step and could contribute to both the control periods and the intervention periods. We used generalized linear models with binomial distribution and identity link to estimate treatment effect adjusting for calendar time (as a categorical variable) and site. We used the generalized equations estimations approach to account for clustering within each clinic and the repeated measures from the same individual over time using compound symmetry covariance structure.

We also explored heterogeneity of intervention effect for different subgroups by testing the interaction between treatment status and subgroup. These groups included site, types of cancer diagnoses, sex as a biological variable, and race and ethnicity (White compared with racial and ethnic minority groups; all races and ethnicities other than White were collapsed into one category, racial and ethnic minority groups for purposes of analysis, because of low numbers of patients of racial and ethnic minority groups). Race and ethnicity were self-reported using categories from the EHR. We assessed race and ethnicity because we prespecified the analysis of White patients compared with patients with ethnic and minority group status. We conducted all analyses using SAS, version 9.4 (SAS Institute Inc).

We used the approach by Hooper et al^33^ to conduct the power analysis. We estimated 4160 patients from 30 oncology practices would be eligible for the study at each time point and a total of 7500 unique patients included in the analysis. With each clinic contributing a mean of 139 patients at each step from the cohort design, the design effect due to clustering was estimated to be 7.9 assuming an intracluster correlation of 0.05, and the design effect due to repeated assessment was 0.12 assuming a cluster autocorrelation coefficient of 0.7 and an individual autocorrelation coefficient of 0.9. These estimates corresponded to an effective sample size (ie, sample size required for individual randomization) of 4405. The study had greater than 90% power to detect a 10% absolute increase of ACP documentation rate (the primary outcome) with a 2-sided significance level of α = .05.

## Results

**Figure 1.**
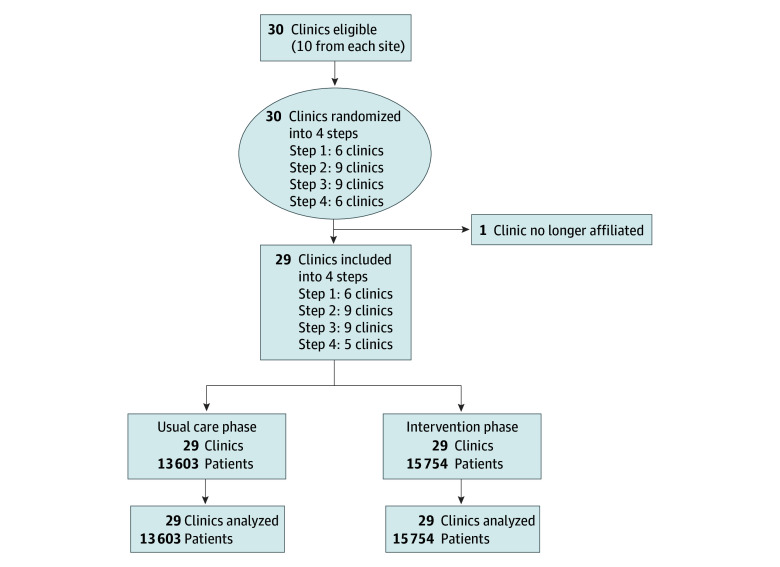
Participant Flow Diagram

During the study period, 13 800 unique patients met the inclusion criteria (8533 contributed to the control periods and 9746 contributed to the intervention periods). With the repeated measurements, these 13 800 unique patients contributed to a total of 29 357 measurements (mean [SD] age, 74.5 [6.6] years; 52.3% men [15 344 of 29 357 measurements] and 47.7% women [14 013 of 29 357 measurements]; 62 American Indian or Alaska Native patients [0.2%], 1035 Asian patients [3.5%], 3433 Black patients [11.7%], 846 Hispanic patients [2.9%], 10 Native Hawaiian or Other Pacific Islander patients [0.03%], 11 multiracial patients [0.04%], 1717 patients of other race and ethnicity, and 22 271 White patients [75.9%]) assessed in any of the 29 ambulatory oncology clinics at our 3 health care systems (Table 1; Figure 1). Due to the randomization order of the clinics, there were more patients with leukemia during the usual care periods and more patients with myeloma during the intervention periods; otherwise, the 2 groups were similar in baseline demographics. Mortality rates among patients from the 3 health care systems were health care system A, 26.2% (1461 of 5570); health care system B, 33.7% (1548 of 4587); and health care system C, 13.4% (959 of 7167).

**Table 1.** Patient Characteristics by Intervention Status

Characteristic	Participants, No. (%)		
	All (N = 29 357)^a^	Usual care (n = 13 603)	Intervention (n = 15 754)
Age, mean (SD), y	74.5 (6.6)	74.6 (6.7)	74.4 (6.6)
Sex			
Male	15 344 (52.3)	6874 (50.5)	8470 (53.8)
Female	14 013 (47.7)	6729 (49.5)	7284 (46.2)
Race and ethnicity			
American Indian or Alaska Native	62 (0.2)	27 (0.2)	35 (0.2)
Asian	1035 (3.5)	479 (3.5)	556 (3.5)
Black	3433 (11.7)	1455 (10.7)	1978 (12.6)
Hispanic ethnicity	846 (2.9)	392 (2.9)	454 (2.9)
Native Hawaiian or Other Pacific Islander	10 (0.03)	3 (0.02)	7 (0.04)
Multiracial	11 (0.04)	10 (0.1)	1 (0.01)
Other^b^	1717 (5.8)	836 (6.1)	881 (5.6)
White	22 271 (75.9)	10 441 (76.8)	11 830 (75.1)
Unknown or missing	818 (2.8)	352 (2.6)	466 (3.0)
English speaking	28 163 (95.9)	13 067 (96.1)	15 096 (95.8)
Health care system			
A	9060 (30.9)	3788 (27.8)	5272 (33.5)
B	8439 (28.7)	4279 (31.5)	4160 (26.4)
C	11 858 (40.4)	5536 (40.7)	6322 (40.1)
Diagnosis type			
Bone marrow transplant	1063 (3.6)	164 (1.2)	899 (5.7)
Brain cancer	649 (2.2)	130 (1.0)	519 (3.3)
Breast cancer	1654 (5.6)	1001 (7.4)	653 (4.1)
Endocrine cancer	804 (2.7)	430 (3.2)	374 (2.4)
Gastrointestinal cancer	4071 (13.9)	1809 (13.3)	2262 (14.4)
Genitourinary cancer	3567 (12.2)	1662 (12.2)	1905 (12.1)
Gynecologic cancer	1403 (4.8)	916 (6.7)	487 (3.1)
Head and neck cancer	338 (1.2)	102 (0.7)	236 (1.5)
Leukemia	3923 (13.4)	3038 (22.3)	885 (5.6)
Lung cancer	1780 (6.1)	652 (4.8)	1128 (7.2)
Lymphoma	2029 (6.9)	307 (2.3)	1722 (10.9)
Melanoma	844 (2.9)	491 (3.6)	353 (2.2)
Myeloma	4036 (13.7)	803 (5.9)	3233 (20.5)
Neuro-oncologic cancer	603 (2.1)	263 (1.9)	340 (2.2)
Sarcoma	1396 (4.8)	883 (6.5)	513 (3.3)
Thoracic cancer	1197 (4.1)	952 (7.0)	245 (1.6)

^a^ From 13 800 unique patients; each patient could be assessed more than once in intervention period and usual care periods.

^b^ Other was the variable used by the 3 health care systems; it did not name specific races or ethnicities.

### Intervention Fidelity

The 3 health care systems (A, B, and C) trained oncologists and advanced practice clinicians in communication skills as each clinic initiated the intervention: health care system A, 63 of 63 (100%); health care system B, 53 of 60 (88.3%); and, health care system C, 59 of 91 (64.8%). Patient video decision aid use in the 3 health care systems included: 516 viewings in health care system A, 2564 viewings in health care system B, and 4561 viewings in health care system C.

### Outcomes

**Figure 2.**
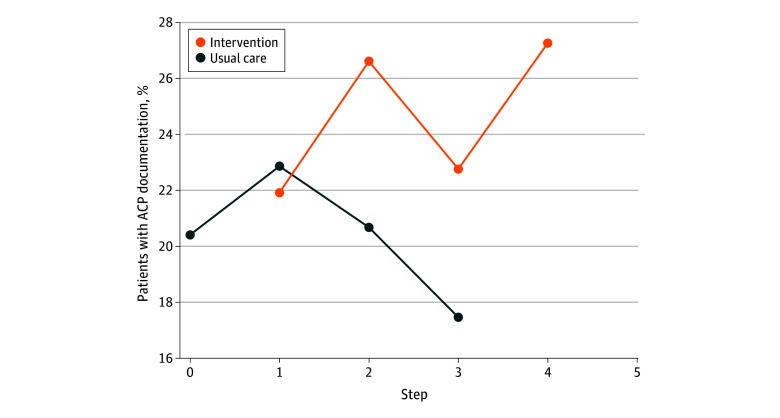
Advance Care Planning (ACP) Documentation by 6-Month Step for the Intervention and Usual Care Periods

The proportion of patients with ACP documentation was greater during the intervention phase compared with the usual care phase (3980 of 15 754 [25.3%] vs 2834 of 13 603 [20.8%]; adjusted rate difference, 6.8% [95% CI, 2.8%-10.8%]; *P* < .001) (Table 2). The ACP documentation rate by step and intervention status are shown in Figure 2.

**Table 2.** ACP Documentation Rate by Intervention Status

Outcome and domains	No. (%)		Adjusted difference, % (95% CI)^a^	*P* value
	Usual care (n = 13 603)	Intervention (n = 15 754)		
Primary outcome				
ACP documentation	2834 (20.8)	3980 (25.3)	6.8 (2.8 to 10.8)	<.001
ACP domains				
Goals of care	2281 (16.8)	3377 (21.4)	6.9 (3.4 to 10.4)	<.001
Palliative care	1287 (9.5)	1517 (9.6)	0.7 (−0.5 to 1.9)	.26
Hospice	724 (5.3)	847 (5.4)	0.3 (−0.4 to 1.0)	.39
Limitation of life-sustaining treatments	1149 (8.4)	1128 (7.2)	−0.1 (−0.7 to 0.6)	.80

Abbreviation: ACP, advance care planning.

^a^ Adjusting for site effect and accounting for clustering within clinic and the repeated measures from the same patient over time.

Documented conversations of goals were greater during the intervention periods compared with the usual care periods (21.4% [3377 of 15 754] vs 16.8% [2281 of 13 603]; *P* < .001) (Table 2). There were no differences between the intervention and usual care periods for palliative care (9.6% [1517 of 15 754] vs 9.5% [1287 of 13 603]; *P* = .26), hospice (5.4% [847 of 15 754] vs 5.3% [724 of 13 603]; *P* = .39), and limitation of life-sustaining treatments (7.2% [1128 of 15 754] vs 8.4% [1149 of 13 603]; *P* = .80).

The intervention effect was similar between White and racial and ethnic minority group patients (rate difference, 4.2% vs 4.6%; *P* = .39) and between male and female patients (rate difference, 4.5% vs 4.5%; *P* = .93), but tended to vary across the 3 health care systems (rate difference: range, −7.4% to 11.1%; *P* = .06) (Table 3). There was also some heterogeneity among the diagnosis types (rate difference: range, −5.0% to 8.6%; *P* = .07): patients with lung cancer, melanoma, gynecologic cancer, gastrointestinal cancer, and breast cancer had a greater than 5% increase in ACP documentation rates; patients with sarcoma, thoracic cancer, lymphoma, brain cancer, endocrine cancer, and myeloma showed smaller increases, and there was no effect on other types of diagnoses.

**Table 3.** Heterogeneity of Treatment Effect

Characteristic	Usual care		Intervention		Difference, % (95% CI)	*P* value
	No.	No. (%)	No.	No. (%)		
Health care system						
A	3788	1238 (32.7)	5272	1333 (25.3)	−7.4 (−9.3 to −5.5)	.06
B	4279	967 (22.6)	4160	1403 (33.7)	11.1 (9.2 to 13.0)	
C	5536	629 (11.4)	6322	1244 (19.7)	8.3 (7.0 to 9.6)	
Race^a^						
Racial and ethnic minority groups	3162	588 (18.6)	3924	895 (22.8)	4.2 (2.3 to 6.1)	.39
White	10 441	2246 (21.5)	11 830	3085 (26.1)	4.6 (3.4 to 5.7)	
Sex						
Female	6729	1468 (21.8)	7284	1914 (26.3)	4.5 (3.0 to 5.9)	.93
Male	6874	1366 (19.9)	8470	2066 (24.4)	4.5 (3.2 to 5.8)	
Diagnosis type						
Bone marrow transplant	164	40 (24.4)	899	145 (16.1)	−8.3 (−15.3 to −1.3)	.07
Neuro-oncology	263	70 (26.6)	340	74 (21.8)	−4.9 (−11.8 to 2.1)	
Genitourinary	1662	449 (27.0)	1905	481 (25.2)	−1.8 (−4.7 to 1.1)	
Leukemia	3038	163 (5.4)	885	48 (5.4)	0.1 (−1.6 to 1.8)	
Head and neck	952	390 (41.0)	245	111 (45.3)	4.3 (−2.6 to 11.3)	
Myeloma	430	83 (19.3)	374	89 (23.8)	4.5 (−1.2 to 10.2)	
Endocrine	803	85 (10.6)	3233	505 (15.6)	5.0 (2.6 to 7.5)	
Brain	102	29 (28.4)	236	83 (35.2)	6.7 (−3.9 to 17.4)	
Lymphoma	307	14 (4.6)	1722	241 (14.0)	9.4 (6.6 to 12.3)	
Thoracic	1809	443 (24.5)	2262	775 (34.3)	9.8 (7.0 to 12.6)	
Sarcoma	130	32 (24.6)	519	184 (35.5)	10.8 (2.4 to 19.3)	
Breast	1001	280 (28.0)	653	255 (39.1)	11.1 (6.4 to 15.7)	
Gastrointestinal	491	107 (21.8)	353	117 (33.1)	11.4 (5.2 to 17.5)	
Gynecologic	652	173 (26.5)	1128	440 (39.0)	12.5 (8.0 to 16.9)	
Melanoma	916	232 (25.3)	487	202 (41.5)	16.2 (10.9 to 21.4)	
Lung	883	244 (27.6)	513	230 (44.8)	17.2 (12.0 to 22.4)	

^a^ All races and ethnicities other than White were collapsed into one category, racial and ethnic minority groups, because of concerns about low numbers of patients of racial and ethnic minority groups. Race and ethnicity were self-reported using categories from the electronic health record.

## Discussion

In this pragmatic stepped-wedge randomized clinical trial among older patients with advanced cancer, an intervention combining patient video decision aids and clinician communication skills training increased the proportion of patients with ACP documentation. Implementation was variable across the 3 health care systems. Clinician communication training was robust across all 3 systems, but implementation of the video decision aids varied.

Older adults with advanced cancer benefit from conversations about serious illness that help oncologists match treatments with patients’ goals. Therapeutic decisions frequently include numerous reasonable options with trade-offs for each; this process requires engaged patients who can express their values and preferences and clinicians who know how to elicit them. Yet for many, these conversations do not take place.

Prior controlled studies^26,29^ have shown that values-neutral video decision aids and clinician communication training, separately, can trigger these conversations and lead to improved ACP documentation. The present trial extends this work by combining these interventions and providing evidence on a health system level of improved ACP documentation for patients with advanced cancer. Although the absolute effect was modest (adjusted estimated rate effect of 7%), when considered across a population, the increase in documentation is both clinically and statistically meaningful. It also uses an intervention that is easily scalable.^26,34^

The entire study period occurred during the COVID-19 pandemic, a time when older patients with advanced serious illness were at increased risk of mortality and during which goals-of-care discussions were critical.^35,36^ The present trial leveraged a combined complementary approach to engage patients and clinicians in goals-of-care conversations by providing patient-facing video decision aids to raise patient awareness along with clinician communication training that prepared clinicians to initiate and be receptive and skilled to have such discussions. Clinical operations across each of the 3 health systems were at times severely tested during the pandemic. Intermittently, clinicians were pulled from clinics and information technology resources were directed at urgent initiatives.^37^ The shift from in-person to telehealth delivery of care also changed the ways patients and clinicians interacted with the health system and how sensitive conversations surrounding ACP were conducted.^37^ Despite these factors, the combined intervention kept ACP discussions present and relevant as the effect of the intervention increased documentation over time compared with the control period.

Even after the intervention, the rate of ACP documentation remained low; however, the mortality rates reported were also lower than expected. Older patients with advanced cancer are at high risk of facing complicated decision-making that is preference sensitive. Additional future studies may need to assess patients who are facing decisions deemed more imminent and critical later in the disease trajectory.

Implementing multicomponent interventions^38^ in large pragmatic trials has been a challenge and our trial was no different. The essential element of our complementary intervention is encouraging patients with video decision aids to reflect on their goals of care prior to or during a clinic visit. This reflection then prompts patients to broach these difficult topics with their clinicians, who are equipped with skills and a clear guiding conversation framework to engage in these discussions. If the intervention is more than the sum of the constituent parts, it would be simplistic to measure the intervention solely in terms of the percentage of clinicians who received communication skills training and the number of video decision aids used by patients.^39^ Reducing complex interventions to their component parts neglects a vital component in the bundled system. However, variations in fidelity can complicate the interpretation of the results.

In our trial, all 3 health systems implemented the communication training, but only 2 of our health systems had robust implementation of the video decision aid component. It is not clear the extent to which this reflects variation in generic implementation factors (eg, leadership, quality culture), features of this intervention model, secular trends (that included a pandemic), or some combination of such factors. However, when there is substantial variation of fidelity across health care systems it remains challenging to interpret the results of the systems that did not implement the bundled intervention with its reinforcing component parts. When health care systems implement only part of a complex intervention, it is unclear whether there are fundamentally different interventions being assessed within the same study.

### Limitations

Our findings must be considered in the context of several limitations. First, stepped-wedge designs present challenges (eg, partial confounding by time) and should be used selectively.^17,40,41^ The ambulatory oncology setting makes contamination a prominent concern, beyond what is tolerated in individual randomization designs.^40,42^ Our study’s robust number of clusters, with disease-based clinics that operate fairly independently, nullify some of the inherent design flaws of stepped-wedge trials.^41^

Second, our study time period occurred entirely during the COVID-19 pandemic. Health care system priorities regarding research resources and staff burden changed daily, impacting the interpretation of our findings. Despite an unprecedented level of competing priorities, the intervention succeeded.

Third, we assessed ACP documentation in the EHR to measure whether the intervention could lead to more and better communication. Prior work has shown that ACP documentation leads to goal-concordant care among decedents, which many view as the ultimate achievement of ACP.^25,43^ Additional studies exploring the relationship between documented preferences and receiving care that is consistent with these preferences are ongoing. Nonetheless, EHR documentation of ACP in clinicians’ notes strongly suggests a shared decision-making encounter that is the criterion standard for patient-centered high-quality care.^24,44,45^ However, to move the field forward, future studies should aspire to assess EHR documentation and actual delivery of medical care at the end of life.

Fourth, we did not assess the quality of the documentation in the entire sample; ongoing analyses of the quality and context of documentation are presently under way. Fifth, we were not able to track video use at the patient level in a manner that allowed direct linkage to either individual patients or to EHR-based outcomes. Future work should follow patient viewing and direct causal linkage of video viewing with the outcomes of interest.

Sixth, the video decision aids in this trial are intended to be support tools used in clinical encounters with clinicians who have been trained in communication skills. Accordingly, we are not able to differentiate the effect of the videos from the communication skills training.

## Conclusions

This randomized clinical trial exploring a combined intervention of patient empowerment and clinician communication skills training found a significant and clinically meaningful increase in ACP documentation rates with a scalable intervention that can be rapidly implemented across large health care systems. This approach offers an innovative paradigm with a clinically meaningful increase in ACP documentation, a widely used quality metric that reflects high-quality patient-centered care delivery.

## Notes

Supplement 1.
Trial Protocol and Statistical Analysis Plan

Supplement 2. **eFigure.** Stepped-Wedge Cluster Randomization Design Scheme of 29 Clinics in Three Health Care Systems **eTable.** Natural Language Processing Keyword Library

Supplement 3.
Data Sharing Statement
