# Supplementary material for: An Intervention to Increase Advance Care Planning Among Older Adults With Advanced Cancer: A Randomized Clinical Trial
Source: JAMA Netw Open. 2025 May 9;8(5):e259150. doi: 10.1001/jamanetworkopen.2025.9150 (PMC12065034; doi:10.1001/jamanetworkopen.2025.9150)
Supplement: Supplement 1. — Trial Protocol and Statistical Analysis Plan [file jamanetwopen-e259150-s001.pdf]

PROTOCOL TITLE:

Advance Care Planning: Promoting Effective and Aligned Communication in the Elderly

PRINCIPAL INVESTIGATORS:

James A. Tulsky

Angelo E. Volandes

10/19/2023

Table of Contents

1.0 Objectives* 3
2.0 Background* 5
3.0 Inclusion and Exclusion Criteria* 7
4.0 Study-Wide Number of Subjects* 8
5.0 Study-Wide Recruitment Methods* 8
6.0 Multi-Site Research* 11
7.0 Study Timelines* 12
8.0 Study Endpoints* 15
9.0 Procedures Involved* 15
10.0 Data and Specimen Banking* 19
11.0 Data Management* and Confidentiality 20
12.0 Provisions to Monitor the Data to Ensure the Safety of Subjects* 24
13.0 Withdrawal of Subjects* 25
14.0 Risks to Subjects* 25
15.0 Potential Benefits to Subjects* 26
16.0 Vulnerable Populations* 26
17.0 Community-Based Participatory Research* 26
18.0 Sharing of Results with Subjects* 26
19.0 Setting 26
20.0 Resources Available 28
21.0 Prior Approvals 29
22.0 Recruitment Methods 29
23.0 Local Number of Subjects 29
24.0 Provisions to Protect the Privacy Interests of Subjects 29
25.0 Compensation for Research-Related Injury 30
26.0 Economic Burden to Subjects 30
27.0 Consent Process 30
28.0 Subject Registration Procedures 30
29.0 Process to Document Consent in Writing 30
30.0 Drugs or Devices 30

REFERENCES

APPENDIX A: Implementation Plan
APPENDIX B: Data Safety Monitoring Plan

1.0 Objectives

The **overall objective** of this application is to reduce the burden of cancer and its consequences for an aging U.S. cancer population. To accomplish this, we propose to plan and conduct a pragmatic stepped wedge cluster randomized trial (SW-CRT) of a Comprehensive ACP (Advance Care Planning) Program among older oncology patients. The Comprehensive ACP Program combines two widely disseminated interventions to assess their impact when used concurrently. One is VitalTalk clinician communication skills training (www.vitaltalk.org) during which the oncology clinicians will practice discussing goals of care with simulated patient actors under the guidance of a trained VitalTalk facilitator. The second is the ACP Decisions video decision aids (www.acpdecisions.org) which are directed to patients and provide education about ACP. The hope is that one intervention activates patients and increases their knowledge and interest in ACP, whereas the other intervention ensures that the clinician who hears these questions has the skills and a cognitive roadmap to manage this conversation in a more expert fashion. The training is five hours, most of which is spent in communication skills work, and the remainder learning how to introduce the videos into one's practice. No patients participate in the training. At the conclusion of each of these trainings, each clinician will be asked to complete a training evaluation. The results of these evaluations will be used to:

- Measure participant reaction
- Measure learning and knowledge acquired
- Measure training effectiveness

This evaluation is comprised of survey questions, it will be anonymous, and poses minimal risk. Therefore, we have requested that consent be waived for clinicians.

Following each training, the newly trained oncology clinic will gain access to the ACP Decisions decision aids which can be introduced to patients at the discretion of their oncology team. Twelve organ-based oncology clinics (e.g., breast oncology, thoracic oncology) from each site will be randomly assigned to an intervention time period – two at a time, twice a year. For example, in Year 1, breast and thoracic oncology will receive the intervention in September and GI and Neuro-oncology will receive the intervention in March. The randomization schedule for all 36 clinics (twelve from each site) will be determined at the beginning of the study and will be created by the study statistician, Dr. Yuchiao Chang at MGH. As a SW-CRT, each clinic will serve as its own control, with the six-month period prior to the intervention serving as the control phase and the six month period after as the intervention phase.

We will recruit patients from organ-based oncology clinics served by three major health care systems: Duke Health (North Carolina), the Mayo Clinic (Minnesota), and Northwell Health (New York). *We hypothesize that a Comprehensive ACP Program of clinician serious illness communication skills training combined with video ACP interventions for patients will improve and sustain rates of ACP*. We will test our hypothesis and achieve our objective through the following:

Specific Aims:

Aim 1: To establish the ***organization, processes, and infrastructure*** necessary to develop all aspects of a pragmatic SW-CRT of the intervention for 29,550 patients 65 and older with advanced cancer in three health care systems, and to ***pilot test*** the intervention study protocol in one oncology clinic in each system. This pilot will include administering the VitalTalk and ACP Decisions training in-person or virtually to all oncology clinicians, identifying 10 patients that meet study eligibility criteria and conducting the patient surveys will be completed either in person, by phone or via an email link to RedCap and video declarations, and extracting the outcome measure data from that health system's electronic health record for all eligible patients in the clinic. The pilot clinics are Head and Neck Oncology at the Mayo Clinic, Sarcoma Clinic at Duke Cancer Institute, and GI Oncology at Northwell Health. Feasibility will be assessed as the ability to successfully complete all aspects of the study protocol, meaning training clinicians at each pilot clinic, documenting usage of videos by clinic patients based on clinic staff report and usage of video access codes, and extracting EHR outcome data by the NLP program and research assistant on 10 patients from each clinic. Advanced cancer is defined as metastatic disease for solid tumors and recurrent or refractory disease for hematological malignancies. If the research team determines that aspects of the protocol need modification to meet the study aims, then such changes will be incorporated into the protocol. However, the interventions themselves (VitalTalk and ACP Decisions) are "off the shelf" products that will not, themselves, be changed. (For a detailed description of the intervention see Appendix A.)

Aim 2: To test the combined effects of a Comprehensive ACP Program on rates of ascertainable quality measures of end-of-life care. We will conduct a SW-CRT across 12 oncology practices in each of the three systems and evaluate the effectiveness of the intervention by comparing the following outcomes among 29,550 ***patients 65 and older with advanced cancer***: Advance care plans completion; Medical orders for resuscitation preferences in the electronic health record; Palliative care consultations; and Hospice use. ***Hypothesis****: A higher proportion of patients in the intervention phase (vs. control) will: complete advance care plans* ***(primary trial outcome),*** *have documented electronic health record orders for resuscitation preferences, be seen in palliative care consultation, and enroll in hospice.*

Aim 3: To characterize detailed patient-centered outcomes, including confidence in future care, communication and decisional satisfaction, and decisional regret in a subgroup of 450 patients 65 and older with advanced cancer, as well as analyses of video declarations from 240 of these patients about their wishes. ***Hypothesis****: Patients in the intervention phase (vs. control) will have improved outcomes.*

IMPACT: Clinician communication training along with video decision support is a practical, evidence-based, and innovative approach to uniformly provide robust ACP. This work has the potential to improve the quality of care provided to millions of older Americans with cancer. Helping oncologists better serve the most frail and vulnerable

older patients by delivering more patient-centered, goal-concordant care could dramatically improve the care of older patients with cancer in health systems.

2.0 Background

SIGNIFICANCE

Epidemiology: Cancer is a common, morbid, and costly condition, especially in patients over the age of 65. Persons over 65 account for 60% of newly diagnosed malignancies and 70% of all cancer deaths. (24) Of the approximately 600,000 adult cancer deaths in 2016 in the United States, 420,000 were patients older than 65. (25) The net Medicare costs of delivering cancer care for older patients are estimated at $21.1 billion per year.(26) The surge in caring for older patients with cancer poses a significant challenge to clinicians and health care institutions.

Advance care planning (ACP) in older patients with cancer needs improvement: ACP seeks to ensure that patients receive medical care consistent with their values, goals and preferences during serious and chronic illness.(10, 11) ACP is the most consistent factor associated with better palliative care outcomes in patients with advanced cancer.(16, 17) The lack of ACP is associated with greater use of aggressive interventions, more terminal hospitalizations, lower hospice use, higher health care costs, and worse family bereavement outcomes.(7, 27-31) Unfortunately, ACP completion in older patients with cancer remains inadequate.(32) Despite passage of the Patient Self-Determination Act in 1991, ACP documentation amongst patients with serious illness has remained consistently low.(33) Furthermore, marked racial and regional disparities persist in ACP documentation for seriously ill patients.(34-39) For the ACP process to lead to optimal decisions, patients require accurate and comprehensible information about their options, and a care setting where communication needs are addressed early in their illness.(12, 13, 27, 40) However, studies show that traditional written and verbal ACP does not effectively inform many patients, and often occurs late in the disease process.(41-46) Patient understanding may be clouded due to pain, medication, or their psychological reactions.(30, 44, 47) Patients' heightened emotional state in response to hearing bad news interferes with cognitive processing and this reaction may be exacerbated when clinicians insufficiently attend to affect.(41, 48-51) Other common barriers to ACP include variable quality of clinician communication, complex relationships between patients, family, and clinicians regarding decision making, and the inability for patients and caregivers to realistically envision future health states.(30, 44, 45, 52-62) Multiple barriers, particularly related to the quality of clinician communication and the ability of patients and families to fully understand possible future scenarios, raise serious concerns regarding the effectiveness of ACP.

Lack of effective ACP leads to burdensome, costly, and often avoidable interventions: Medical technology enables an extraordinary array of possible interventions for older people living with cancer. However, particularly in the setting of advanced disease, the burdens of treatment may outweigh its beneficial effects. Expected clinical complications

at the terminal stages of advanced cancer include respiratory failure, inability to eat, and serious infections. (63) Patients' individual values and goals determine whether to initiate mechanical ventilation, insert a feeding tube, or attempt cardio-pulmonary resuscitation (CPR). Without the presence of an ACP discussion, the default approach is to perform all invasive interventions. Research suggests that these “rescue” interventions are inappropriately used even when not desired.(1, 38, 64) Despite the mounting evidence suggesting that patients with serious illness prefer palliation in the advanced stages of cancer, they often receive burdensome interventions and sub-optimal control of pain and other distressing symptoms, particularly in the absence of high-quality ACP.(1-4) The converse is true for patients who have had documented decisions about their end-of-life care.(16-18, 34, 65-67) ACP is necessary to avoid unwanted and potentially unnecessary interventions at the end of life; high-quality ACP increases patient safety by ensuring that patients receive effective medical care that meets their goals at the end of life.

Clinician communication training and video decision support improves ACP: The traditional approach to ACP, which primarily relies on ad hoc verbal descriptions of hypothetical clinical situations and treatment choices, is limited because complex scenarios are difficult to envision, provider information is inconsistent, and verbal explanations are hampered by literacy, emotional and language barriers.(42, 68, 69) Patients often do not fully understand the choices presented in advance directive legal documents, and the quality of communication about these choices is suboptimal.(70) Over the past few years, investigators have recognized the shortcomings of prior efforts and have developed new interventions to better facilitate ACP.(10) The Comprehensive ACP Program proposed for this study focuses on training patients and clinicians on communication. For patients, video decision aids to better educate and inform decision making are commonly used, and the ACP Decisions video based tools are the best example of these. These videos attempt to overcome literacy barriers and to present potential scenarios with a sense of reality lacking in verbal descriptions. ACP Decisions has also introduced the ability of patients to record themselves on video stating what is most important to them when they have a serious illness and their choices for medical care. For clinicians, the VitalTalk program is the most widely disseminated teaching method that focuses on patient-centered communication skills training. Prior work, including several RCTs, supports the efficacy of the ACP Decisions videos and the VitalTalk program in patients with advanced cancer. (19, 20, 22, 23, 71-83) This application represents the first Comprehensive ACP Program combining two well-tested, evidence-based, and complementary interventions, that treat patients and clinicians as equal stakeholders by providing both with the communication skills and tools needed to optimally engage with ACP.

A pragmatic, stepped wedge, cluster randomized trial (SW-CRT) is well-suited to evaluate a Comprehensive ACP Program intervention in older patients with cancer: SW-CRTs are increasingly used in health services research, and there are several notable advantages of this design for testing communication interventions in patients with cancer.(84) With the oncology clinic as the unit of randomization, the contamination that can occur when randomizing individuals within clinics is avoided. The stepped wedge

design is also practical and considered the design of choice when it's logistically impractical to simultaneously roll out the intervention to half the clusters (as in a parallel cluster RCT). In addition, the interventions being tested will be implemented at the clinic level and individual consent procedures may be unnecessary for low risk studies. To date, most randomized trials conducted with older patients with serious illness involved few facilities and evaluated the effects of interventions under ideal circumstances (i.e., explanatory trials).(85-88) Pragmatic trials, which intend to determine the effects of interventions under usual conditions, are a next critical step in research involving older patients with cancer.(89, 90)

Summary of significance: The significance of this study rests on five premises: 1. Cancer is a common, morbid, and costly condition for patients over 65; 2. Older patients with advanced cancer often receive aggressive and costly interventions that may be of little clinical benefit and inconsistent with their goals; 3. An opportunity exists to promote preference-based, higher quality, and more informed ACP with patients with cancer through a Comprehensive ACP Program earlier in their disease trajectory; 4. An intervention comprised of clinician communication training and a suite of ACP video tools presents a promising, scalable, and efficacious approach that can be implemented on a system-wide level in a flexible manner; and, 5. Oncology clinics offer an ideal setting for a pragmatic trial. Taken together, this work has the potential to improve the quality of care provided to millions of older Americans with cancer and enable future pragmatic trials in this increasingly important patient population.

3.0 Inclusion and Exclusion Criteria

Physician, Staff Eligibility: Any staff member identified by the site-PI (Drs. Zafar, Pollak, Tilburt, Loprinzi, Martins-Welch, and Carney) who is affiliated with the clinic.

Clinic Eligibility: Three health care systems are participating in the study, each contributing 13 oncology clinics (1 for UG3 year; 12 for UH3 years): Duke Health, Mayo Clinic, and Northwell. Clinic eligibility criteria include:

- More than one oncologist
- Serve a patient population that is at least 30% aged 65 or older
- Disease-based oncology clinic

Subject Eligibility (for the patient surveys): Any patient affiliated with one of the study clinics who speaks English and is aged 65 or older with advanced cancer is eligible for participation. Advanced cancer is defined as metastatic disease for solid tumors and recurrent or refractory disease for hematological malignancies. There are no exclusions based on gender, race, or ethnicity.

Patients will be identified as having advanced cancer by identification from clinical ICD codes. Use of ICD codes has been studied extensively at Dana-Farber Cancer Institute (DFCI), and it captures enough patients with advanced cancer with high specificity for

outcome assessment without systematic bias towards intervention or control periods. During the UG3 year, our site-PIs will identify patients aged 65 or older with advanced cancer using the EHR and ICD codes and assess their outcomes using the EHR dataset. They will then de-identify (10 patients at each site; 10 patients * 3 sites = 30 patients' EHR records) actual medical record information about these individuals to validate their diagnoses and outcomes. For disease types where the ICD identification has not been validated or tested, such as liquid tumor clinics, we will identify alternative means for selecting advanced cancer patients, such as age and disease related variables in the EMR.

- We will not be including adults unable to consent
- We will not be including individuals who are not yet adults (infants, children, teenagers)
- We will not be including pregnant women
- We will not be including prisoners

4.0 Study-Wide Number of Subjects

Over the four years of the UH3 phase of the trial, we will study data from 29,550 patients with advanced cancer aged 65 or older for our primary (completed advance care plans) and secondary (resuscitation preferences, palliative care consultations, hospice use) outcomes. These data will be obtained from the EHR. In addition, we will also recruit 450 eligible patients (150 patients from **each** of our three sites broken down into 75 patients during the control phase and 75 patients during the intervention phase) to conduct a survey either in person, by phone or via an email link to REDCap), for our secondary patient-centered outcomes (confidence, satisfaction with physician communication, patient decisional satisfaction and regret). From among this sub-group we will engage 240 participants (80 from **each** of our three sites broken down into 40 patients during the control phase and 40 patients during the intervention phase) in an activity to film video declarations of their preferences. The total number of patients will be 30,000. Again, our overall accrual goal is 30,000where 29,550
of these patients, the vast majority, will be included only for medical record review. 450 participants will complete an in-person survey and, among that group, 240 participants will complete a video declaration.

5.0 Study-Wide Recruitment Methods

Recruitment of patients for survey:

Methods:
The recruitment methods will only apply to those patients that we will individually survey, 450 patients over the course of 3.5 years. Patients with advanced cancer (N=450) will have an in-person survey over the course of 42 months of recruitment as indicated above (each step = 6 months). Surveys will be evenly distributed among the three health care systems (i.e., 150 patients from each system), and will include an even number of surveys of patients randomly chosen to clinics in the control phase (i.e., 75 patients

Patient Surveys Total by Step	6 Months Prior	Step 1	Step 2	Step 3	Step 4	Step 5	Step 6
**Duke Health - Target**	12	25	25	25	25	25	13
**Duke Health - Actual**							
**Mayo Clinic - Target**	12	25	25	25	25	25	13
**Mayo Clinic - Actual**							
**Northwell Health - Target**	12	25	25	25	25	25	13
**Northwell Health - Actual**							
**Target Total by Step**	36	75	75	75	75	75	39
**Actual by Step**	0	0	0	0	0	0	0
**Target - Cumulative**	36	111	186	261	336	411	450
**Actual - Cumulative**	0	0	0	0	0	0	0

surveyed in control phase) and in the intervention phase (i.e., 75 patients surveyed in intervention phase). For each clinic, control patients will be randomly chosen and surveyed during the 6 months prior to the intervention, and intervention patients will be randomly chosen and surveyed during the 6 months after the intervention.

Patients will not be surveyed more than once. Each clinic will have their control surveys conducted six-months prior to the intervention, and their intervention surveys in the step thereafter. Research staff will exclude any participant that was surveyed during the control period to ensure that the participant is not surveyed during the intervention period.

From among this group of 450 participants, we aim to conduct the video declaration of preferences activity with 240 patients. The 240 patients will be evenly recruited among our three health care systems (80 patients per system). Half of patients will be recruited during the control period (N=40) and half of patients (N=40) will be recruited during the intervention period as indicated below.

Video Declarations Total by Step	6 Months Prior	Step 1	Step 2	Step 3	Step 4	Step 5	Step 6
**Duke Health - Target**	7	12	14	14	12	14	7
**Duke Health - Actual**							
**Mayo Clinic - Target**	7	14	12	14	14	12	7
**Mayo Clinic - Actual**							
**Northwell Health - Target**	6	14	14	12	14	14	6
**Northwell Health - Actual**							
**Target Total by Step**	20	40	40	40	40	40	20
**Actual by Step**	0	0	0	0	0	0	0

**Target - Cumulative**	20	60	100	140	180	220	240
**Actual - Cumulative**	0	0	0	0	0	0	0

During the control and intervention phases of the trial respectively, an RA will use the EHR to identify potential participants with advanced cancer being seen in the clinic. The RA will review a list of scheduled patients two weeks prior to their clinic visit. Only returning patients (i.e., not new consults) who speak English and are known to the clinicians will be considered. Applying an International Classification of Diseases and Related Health Problems (ICD-10) code system algorithm to the EHR, the RA will initially identify potential participants aged 65 or older with advanced cancer
The RA will use a broad selection of ICD-10 codes for advanced cancer, to be as inclusive as possible in the screening process. The RA will then review the EHR to verify that the patient meets eligibility criteria. To conduct this screening procedure, a waiver of individual authorization for disclosure of personal health information (HIPAA) will be obtained from each site. For those patients meeting all the criteria for advanced cancer, the RA will randomize the order of scheduled patients and then start at the top of this list and go down until fulfilling the enrollment target. The RA will then contact the patient's primary oncologist by email to solicit his/her opinion as to whether the patient is appropriate to approach for participation. Once a potential participant is identified, and the oncologist has approved, a letter outlining the project will be mailed or emailed using the institution's secured email system to the patient prior to the clinic visit.

On the day of his/her clinic visit, or by phone the day before the visit, the RA will make contact with the patient to further explain the study and obtain verbal informed consent for the survey. If the RA conducts the survey over the phone or if the participant completes it using an email link to REDCap, they will email using the institution's secured email system or mail the IRB-approved detailed information sheet with all elements of consent that we would hand them if we were to do so in person.

For those who complete the survey in person the RA will explain the video declaration portion of the trial and obtain written informed consent. Participants who we will not see in person and, for whom, obtaining signed consent is logistically challenging and burdensome will be presented with all required elements of consent via a previously approved Detailed Information Sheet ViDec and a consent discussion will occur, but the consent form will not be signed. Only those participants who can understand the aims of the project, what their involvement entails, that participation is voluntary, and the risks and benefits of participation will be eligible for the study.

"Broadcast notifications":

We will be providing any potential patients (participants) with the opportunity to opt out of this project entirely (i.e., to have their deidentified data from the EHR not included in our main analysis). We have created "broadcast notifications" in the form of a poster in the clinic/patient areas giving brief commentary about the trial and their choice to

participate or not to participate in having their data included in our analysis. One of our health care systems routinely does this for all patients at registration (Mayo Clinic), and we have extended these "broadcast notifications" to our other two sites.

6.0 Multi-Site Research

COMMUNICATION

Communication for the trial is multi-tiered. The committees and working groups maintain regular meeting calendars and have representatives who function as liaisons for other groups within the study. Meeting agendas and minutes are prepared and posted on the trial UG3/UH3 internet site, which functions as an ongoing venue for document sharing and communication. The majority of meetings are held via teleconference through WebEx or Zoom, as well as periodic in-person meetings.

Working Groups:

Topic-specific groups (e.g., Executive Committee, Regulatory and Ethics Working Group) meet monthly or as needed for the duration of the study. Working group leaders communicate relevant actions/information resulting from working group meetings at the monthly Steering Committee meetings.

Each "working group" is tasked with reporting, any issues to the larger group. The Executive committee will report about any regulatory concerns. The specific designated "Working Groups" will oversee dissemination of interim results and information about the closing of the study.

DATA STORAGE AND SECURITY

Data Management:
We are working with the DFCI-based "Survey & Data Management Core." Data will be managed by the Data Standards and Quality Working Group led by Dr. Lakin. The RA and local champion at each site, where data is being collected, will extract data every six months (i.e., the length of each step) from the EHR and surveys. Each site will maintain and adhere to the process and procedures for the protection of human subjects and protected health information (PHI) for their covered entities. All data collected by the RAs will be stored in password protected servers. Participant identifiers will be kept in separate password protected files and a third linking file will be maintained. The linking file will also be password protected, access will be minimized, and a logging feature will be used to identify each user and instance of use. Only the minimum amount of PHI necessary will be collected from study participants. Data from each of the clinical sites will be transmitted via secure, institutionally approved methods to DFCI for data management and to Boston Medical Center (BMC) for qualitative analyses and MGH as well. The BMC Research Assistants, under the direction of Dr. Quintiliani, will produce

written transcripts of the videos (including information on non-verbal cues) and may also assist in coding the transcripts and grouping/summarizing the codes into themes for analysis.

All information in the REDCAP database will be indexed by subject identifier only, so that even if the database server is compromised subjects cannot be identified, thus maintaining the privacy of their information. Also, assurance of confidentiality of information will be made to all subjects. Data will be handled with the same confidentiality accorded to patients' medical records. Specific procedures protecting subject confidentiality will be as follows:
1. ID number only will be placed on electronic (or paper) study forms or records on which data are collected and/or stored.
2. Access to data files will be secured with a password-filing system (that logs entry) and is restricted to authorized staff only.
3. Necessary hard-copy records containing study data of any type will be kept in locked files.
4. Master lists linking subject information with ID number will be numbered consecutively and prepared before data collection (to ensure accurate accounting). These lists will be kept locked, in duplicate, with access only by the PIs and the other investigators.
5. All project staff will sign an oath of confidentiality to ensure their understanding of the terms of confidentiality required. They will be trained in specific procedures to ensure confidentiality.
6. Sign-out procedures for all access to data files will be strictly enforced.
7. All reports and publications will preserve participants' anonymity.

We will use of Dropbox for Business as the central repository for storing all of the fixed and NLP data that the sites send to us which will allow our data team to store data, documentation about data and code used to analyze the data in a platform that best compliments their workflow. DBFB is HIPAA compliant approved for use by DFCI and will only be accessed by DFCI study staff. Northwell staff do not have the ability to upload any files to Dropbox for Business so they will upload their Videos to OneDrive and provide project staff at DFCI access so that they can then upload those videos to Dropbox For Business.

Long-term storage of Data:

Data stored on the DFCI server will reside there only for the periods they are required to be there for study usage. Data will be securely removed from these servers on a per-item basis. Removed data will be securely transferred to DFCI long-term servers for storage.

7.0 Study Timelines:

Study Timeline:

During the UG3 year beginning on July 1, 2018, we will finalize the list of all 39 oncology clinics and recruit 3 pilot clinics (one from each health care system) by month 2 (August 2018). The remaining clinics (i.e., 36) will be randomized in pairs for the remaining six steps of implementing the intervention. By month 3 (September 2018), we will begin baseline data collection for the three pilot clinics using their EHRs. We will refine and finalize the intervention during months 3-4 and begin trainings (October-November 2018) in the three pilot clinics. By month 5-6 (November-December 2018) implementation of the intervention at the three pilot clinics will begin and intervention-period data will be collected at the three pilot clinics. By month 10 (April 2019), data extraction and cleaning, and measurement validation will be performed from the first set of intervention data from the three pilot clinics. During month 10, a UG3 report and UH3 proposal will be submitted to the NIA. Exit interviews of staff will be conducted during months 11-12 (May-June 2019). We will create video declarations for 10 patients at each clinic, for a total of 30 during the first year.

During months 1-3 of the UH3 year beginning on July 1, 2019 and continuing every six months (for a total of 6 steps), we will randomize 2 oncology clinics in each of our three health care systems to receiving the intervention (Figure 1).

**Figure 1**	UG3	UH3					
Cluster	Baseline	1m	7m	13m	19m	25m	31m
1, 2							
3, 4							
5, 6							
7, 8							
9, 10							
11, 12							

The implementation period covers at least 6 months in each clinic. Data extraction and merged database creation will start during the UG3 year and extend through approximately 6 months past the end of the implementation phase of the last step; ensuring full follow-up of outcomes. The final dataset will be complete and analyses will begin in the last six months of the trial. Manuscript preparation and dissemination of the trial results will occur during the final three months.

The ACP-PEACE trial (**P**romoting **E**ffective **A**dvance **C**are planning in the **E**lderly) is a stepped-wedge cluster randomized trial (SW-CRT) of a combined advance care planning (ACP) program consisting of in-person clinician communication skills training and patient-facing video decision aids in 36 oncology clinics across three sites. In the original design, after a baseline period of six months, a pair of intervention clinics at each of our three sites was to be trained in the intervention. The training would be repeated every six months (6 months = 1 step) for six steps (6 steps x 2 clinics/step x 3 sites = 36 clinics). (See Figure 1.) The primary outcome is ACP completion rates in advanced cancer patients aged 65 years or older.

Figure 1. Original Stepped-Wedge Design of ACP-PEACE

		STEPS					
Clinic	Baseline	1	2	3	4	5	6
1, 2							
3, 4							

5, 6							
7, 8							
9, 10							
11, 12							

The ACP-PEACE trial successfully completed the baseline data collection and Step 1 training (2 clinics per site x 3 sites = 6 intervention clinics). However, during Step 2, COVID-19 spread throughout the country interrupting our stepped-wedge design in two respects. First, the team was unable to conduct the in-person training for the Step 2 intervention clinics because of travel and social distancing restrictions. Second, motivated by the disproportionate risk to cancer patients from COVID-19 and the higher mortality rates among their older patients (i.e., the cohort of patients in the ACP-PEACE trial), oncologists in both control and intervention clinics increased ACP activities outside of the trial, thus changing the baseline ACP rates (our primary outcome) in both control and intervention clinics (i.e., the "COVID-19 effect").

Following the recommendation of the NIH Collaboratory Statistics Core, we propose using our original Step 2 period as the new baseline for the remaining 30 clinics and to "restart" the trial (See Figure 2.) This would allow us to train the remaining 30 control clinics over four steps (i.e., every six months) keeping the trial completion on time.

Figure 2. Proposed New Stepped-Wedge Design of ACP-PEACE with COVID-19 Effect

Clinic	Baseline	1	2	3	4	5	6
1,2							
			Baseline	1	2	3	4
3,4,5							
6,7							
8,9,10							
11,12							

Intervention	Covid-19
no	no
no	yes
yes	no
yes	yes

The data we collect from the 36 clinics will allow us to address the changes due to COVID-19. In addition to the study's original aims (which remain the same), we are now adding three new specific aims:

Aim 1: Examine the ACP Program intervention effect prior to COVID-19: This will be done using the data from the 6 clinics from our original Step 1. We will estimate ACP rates prior to the intervention (original baseline) and after the intervention (Step 1).

Aim 2: Examine the intervention effect post COVID-19: This will be done using the data from the remaining 30 clinics. We will have the stepped-wedge design starting in the original Step 3.

Aim 3: Examine the COVID-19 effect: This will be done using the data from the remaining 30 clinics. We will estimate the ACP rate prior to COVID-19 from the original baseline and Step 1, and the ACP rate post COVID-19 using the original Step 2 data. Additionally, we can examine the time trend after the new baseline to see how the COVID-19 effect diminishes over time from control period clinics.

8.0 Study Endpoints

Primary & Secondary Endpoints:

We will conduct a SW-CRT of the effect of a Comprehensive ACP Program on rates of ascertainable quality measures of end-of-life care. Our primary outcome is documentation of ACP activity as determined through natural language processing (NLP) of EHR records confirmed by human coders. Discussions of goals of care, completion of advance plans, and changes of resuscitation orders will all count in this composite measure. Secondary outcomes are resuscitation preferences, palliative care consultations, and hospice utilization. These outcomes will be ascertained from the records of 29,550***patients*** across oncology practices in the three enrolled health systems.

Secondary safety endpoints.

There is a risk of patients becoming uncomfortable with discussing these topics.

To minimize the risks of patients becoming uncomfortable, study staff will emphasize that they can take a break if they need to, or can quit the study all together if they decide they no longer want to participate. If patients become distraught and they wish to stop, we will notify their nurse and attending physician. We will then follow any recommendations they have for obtaining support as needed. If patients become distraught during the second video declaration and we are in their home, we will contact their attending physician to explain the situation and follow their recommendations for obtaining support as needed.

9.0 Procedures Involved

Data Collection Protocol:

Patients: Baseline data collection of the 36 randomized clinics will begin by month 10 of the UG3 phase. Data Collection will continue and occur throughout the UH3 phase of the trial. Our primary (advance care plans) and secondary (preferences for resuscitation, palliative care consults, hospice use) outcomes will be abstracted from the EHR and the local tumor registry.

A group of older patients with advanced cancer (N=450) will have a survey over the course of the 3.5 years of recruitment. From among this group of 450 participants, we aim to conduct the video declaration of preferences activity with 240 patients.

Based on our prior studies, data collection is estimated to take no longer than 30 minutes per patient (15 minutes for survey questions, 15 minutes for video declaration) and will be conducted as convenient for the patient. The relatively brief interviewing time (30 minutes) in which the survey is conducted should assure completion of the interview

without burdening participants who have advanced cancer. We do not foresee the additional time to complete the survey to be a barrier to successful recruitment and completion of the protocol.

Participants will be provided written copies of the questions to follow along during the interviews, or the questions will be emailed if the survey is conducted over the phone or if the participant completes it using an email link to REDCap. For the subgroup of patients who complete the surveys, the RA will also ask participants to complete a video declaration. For those participants that agree to the video declaration, they will proceed with recording of their video declarations.

The RA can provide these patients with the questions before they are videotaped, either during the consent process, and before the recording the video or after they have consented, and before the recording the video. Participants who we will not see in person and, for whom, obtaining signed consent is logistically challenging and burdensome will be presented with all required elements of consent via a previously approved Detailed Information Sheet ViDec and a consent discussion will occur, but the consent form will not be signed.

To assist participants in creating a video of themselves describing their ACP preferences, the RA will begin by reading a standardized introduction to the subject: "Imagine you weren't able to talk to your doctors or family because you were very sick. We would like you to make a video about your preferences for medical care so your doctors and family can understand what's most important for you. Please try to be specific. For example, if you could not eat by mouth would you want doctors to insert a feeding tube? Or if you could no longer breathe on your own, would you want the doctors to place you on a breathing machine – a mechanical respirator? I'll show your video to you when you are done. If you aren't happy with it, you can record it again. Do you have any questions for me before we get started?" The introduction will be pilot tested during the UG3 phase and modified as needed. The RA then uses the camera on the tablet (e.g., iPad) as a video device to record the subject. The tablet will be situated so that the subject is not viewing themselves on screen while they are talking, to lessen feelings of self-consciousness while recording. The RA will provide prompts as needed, such as if the person is exhibiting extreme hesitation in not knowing what to say, or if the person does not address their wishes regarding CPR or intubation. In these cases, the RA will ask some questions to facilitate the video process. When the recording is complete, the RA will play the video for the subject to see if they feel it accurately represents their preferences and if they would like to re-film their video declaration. (In our preliminary study, one out of 15 patients wished to re-record their video. However, we feel it is important to allow participants this flexibility when needed. Patients are able to re-film as many times as they want.)
To provide patients with a comfortable setting for completing their video declarations, we will offer patients the option of completing these declarations during RA home visits.

We will discuss the option of sharing the video with the patient. If they are interested, we will ask the participant how they would like to share their video. There are four choices: 1. We can put the declaration video on an encrypted flash drive which is password protected and provide the flash drive to the participant; 2. We can post the declaration video on a website called Dropbox for Business. The participant would be provided a web link to view the video online. Dana-Farber has more privacy control over this site and can remove the video at any time; 3. We can put the declaration video on an unencrypted flash drive which is not password protected and provide the flash drive to them; or, 4. We can post the declaration video on a YouTube unlisted video setting under the study's YouTube account and provide the web link to the participant. An unlisted video can only be seen and shared by a web link. The unlisted video should not be available on YouTube's search results or for people who do not have access to the web link.

<https://support.google.com/youtube/answer/157177?co=GENIE.Platform%3DDesktop&hl=en<https://support.google.com/youtube/answer/157177?co=GENIE.Platform%3DDesktop&hl=en>>>

There are times when the research team would like to share participants' videos with their colleagues, in scientific presentations or to train study staff. We will ask participants if they would be comfortable in sharing their video publicly for purposes like this. The risk is that the video could be widely shared, depending on the venue. If a participant begins to feel any distress and changes their mind about sharing the video publicly, they can let us know and we will stop sharing it publicly.

We will discuss sharing the video with the patient. We will ask the participant how they would like to have their video (e.g., uploaded to YouTube, DropBox, USB drive), and with whom they would like to share it with (e.g., oncologist, primary care provider, family).

Data collected for long-term follow-up:

Performance of NLP

Dr. Lindvall's lab at DFCI has published several studies on the use of Natural Language Processing (NLP) to capture documentation of ACP in clinical notes.(91 – 93) Compared to standard medical record review her team has achieved a sensitivity 97% - 99% and specificity 85% - 100% at the note level. At the patient level, both sensitivity and specificity are close to 100%. Patients accrue hundreds to thousands of notes which makes standard medical review not feasible, and this method dramatically increases efficiency and sensitivity.

Advances in computer science techniques such as NLP allow for efficient and accurate analysis of the free-text from clinical notes in order to detect our primary outcomes. The

increasing use of EHR combined with computational advances in NLP can accurately quantify our primary and secondary outcomes.

Over the course of the study, we will use NLP to analyze outcomes documented in the clinical notes for all of the patients aged 65 or older (29,550)

The below procedures will be completed at each health care system by their respective site-Investigators. No patient identifiable information will leave the HIPPA-secure firewall of each health care system.

Each site-Investigator will use NLP to identify the following primary and secondary outcomes.

**Primary outcome**	**Domain**	**Definition**
Advance Care Planning	Goals of care	Conversations with patients or family members about the patient's goals, values, or priorities for treatment and outcomes. Includes statements that conversation occurred as well as listing specific goals.<br><br>OR<br><br>Documentation advance care planning was discussed, reviewed, recommended, or completed.
Advance Care Planning	Code status limitations	Conversations with patients or family members about preferences for limitations to cardiopulmonary resuscitation and intubation.
Advance Care Planning	Palliative Care	Documentation that specialist palliative care was discussed, patient preferences regarding seeing palliative care clinician.
Advance Care Planning	Hospice	Documentation that hospice was discussed, prior enrollment in hospice, patient preferences regarding hospice, or assessments the patient did not meet hospice criteria.

**Secondary outcomes**	**Definition**
Surrogate Decision Maker	Documentation of a surrogate decision maker.
Video	Video support tool was recommended or viewed.

Data will be abstracted from the EHR data repository (see above tables) at each health care system. Demographic information and baseline characteristics relevant to general oncology will be collected. Analysis of free text regarding patient preferences and goal-of-care conversations will be extrapolated using computational methods.
Individual variables have been selected as they are basic demographic and baseline characteristics of study participants that are relevant to general oncology patients (disease location, stage, performance status). The primary and secondary outcomes are widely used as measures of end-of-life care in the cancer population.

The Investigators at each health care system will use computational methods (NLP) to identify our outcomes such as documentation of goals of care and patient preferences.

Once these variables have been abstracted from the health care system's EHR, the data will be collected for each patient and the patient will be given a random unique identifier. A cross-walk of the unique identifier and the identifiable information of the patient will be securely stored at the health care system and not transferred to DFCI.

The deidentified data for each patient will then be sent to DFCI and MGH for further analysis.

Only researchers listed on this IRB will be able to access the data at each health care system. The risks will be minimal as the data will be stored and analyzed on the HIPAA secure cluster at each health care system. None of the data will be stored in paper form. The data and identifiers will be kept for three years on the HIPPA secure cluster computer at each health care system. After the three years, the data will be permanently destroyed.

Quality Control to the NLP

We will do a 1-time random sample of 10% of patients participating in the study over a 6-month period at each site. This is a way to validate what everybody is doing the process at each study site, that what they are extracting from the EHR for the NLP analysis is accurate. A visiting core staff member will go to the site. The RA at the site will select a random sample of 10% and, then in real time, pull up all the EHR notes from those 10% of patients. They will, and then compare the notes that they originally pulled for the NLP analysis, to the notes that the staff member and RA pulled on the visit. We define high quality as >90% accuracy between pulled EHR data and the 'live' EHR. If the accuracy is lower, then we will investigate reasons for the discordance.

10.0 Data and Specimen Banking

N/A

11.0 Data Management and Confidentiality

DATA ANALYSES

Aim 2 (UH3): To test the combined effects of a Comprehensive ACP Program on rates of ascertainable quality measures of end-of-life care. We will conduct a SW-CRT across 36 oncology practices in the three systems and evaluate the effectiveness of the intervention by comparing the following outcomes among 29,550***patients aged* 65 or over with advanced cancer**: ACP documentation; Medical orders for resuscitation preferences in the EHR; Palliative care consultations; and Hospice use. ***Hypothesis****: A higher proportion of patients in the intervention phase (vs. control) will: complete ACP documentation* **(primary trial outcome),** *have documented EHR orders for limitations on resuscitation, be seen in palliative care consultation, and enroll in hospice.*

With the stepped wedge design, the outcomes during the intervention (exposed) periods will be compared to outcomes during the control (unexposed) periods. We will summarize both clusters and patients' characteristics by exposure status to examine potential selection biases and lack of balance. We will use a generalized linear mixed model with a logit link to compare the rate of advance care plans between intervention and control periods. Our basic model is as follows: $logit(Y_{ijk}) = \mu + \alpha_i + \beta_j + \gamma_k + X_{ij}\theta$
where Y_{ijk} denotes the response from individual *k* at time *j* from cluster *i;* α_i is a random effect for cluster *i*; X_{ij} is the treatment indicator for cluster *i* at time *j; and* θ is the treatment effect. Since intervention occurs over time, the proportion of clusters exposed to the intervention gradually increases. We will include step as a fixed effect (β_j) in the model to adjust for the potential confounding factor from calendar time. We added a random effect for individuals *(*γ_k) to account for the repeated measures since patients are likely to be present in multiple periods.

We will entertain several extensions to the basic model. (1) In the case that time effect might not be the same for all clusters, we will change the term for time effect from a fixed effect β_j to a random effect β_{ij}; (2) In the case of treatment effect heterogeneity, we will either change the fixed effect θ to a random effect θ_i or change the fixed effect θ to $\theta_{(s)}$ which allows different treatment effects for different strata; (3) As the intervention might not become effective immediately after it is introduced, we will consider either allowing X_{ij} (treatment indicator in cluster *i* at time *j)* to be a fractional number between 0 and 1, or changing $X_{ij}\theta$ to $X_{ijl}\theta_l$ to account for the number of steps since the intervention was introduced; (4) If necessary, we will add additional terms $\gamma_1 \boldsymbol{Z}_{ijk}$ and $\gamma_2 \boldsymbol{W}_{ij}$ to the model, where ***Z*** and ***W*** represent vectors of patient and cluster characteristics that could be predictors of outcomes, to adjust for any imbalance from clusters and patients' characteristics. Note that the index *j* in these matrixes allows us to include time-varying covariates. In addition, we will include one of the $\boldsymbol{Z}_{ijk}$ to be defined as “time since recruitment/enrollment” to account for the (possible) increase in likelihood of completing an ACP as disease progresses, independent of any intervention effect.

In a secondary analysis, we will explore the heterogeneity of intervention effect for different subgroups such as health care system, sex as a biological variable, race/ethnicity (white vs. non-white), or different types of cancer diagnoses (see model extension [2] above). We will also take advantage of the stepped wedge design to compare outcomes from the exposed and unexposed periods within each cluster. We will use the same approach for our secondary outcomes (e.g., resuscitation preference) with the exception that the number of palliative care consults will be considered as a Poisson variable and modeled with a log link.

Sample Size Estimates are based on the primary study outcome: ACP documentation among patients aged 65 or over with advanced cancer. We used the Hooper et al.(85, 86) approach to conduct the power analysis. We originally estimated close to 5,000 patients from 36 oncology practices are eligible for the study at each time point and approximately 20% are new patients at each step. With 7 time points (baseline plus 6 steps), we anticipated a total of 11,000 unique patients will be included in the study. With the modified design, we estimate 4,160 patients from 30 oncology practices are eligible for the study at each time point and a total of 7,500 unique patients will be included in the stepped-wedge design analysis. With each clinic contributing an average of 139 patients at each step from the cohort design, the design effect due to clustering is 7.9 assuming an intra-cluster correlation of 0.05, and the design effect due to repeated assessment is 0.12 assuming the cluster autocorrelation coefficient is 0.7 and the individual autocorrelation coefficient is 0.9. These estimates correspond to an effective sample size (i.e. sample size required for individual randomization) of 4,405. For the repeated cross-sectional design, each clinic will contribute an average of 23 new patients at each step, and the effective sample size is 1,628 with the same assumptions on intra-cluster correlation and cluster autocorrelation. Preliminary estimates indicate the rate for ACP documentation (the primary outcome) ranges from 15% to 30% for the control periods, which requires an effective sample size of 500 to 954 for detecting a 10% absolute increase in our primary outcome with a two-sided significance level of 0.05. Therefore, the study will have more than 90% power for either analysis using the open cohort with repeated measures design or the repeated cross-sectional design.

Aim 3 (UH3): To characterize detailed patient-centered outcomes, including confidence in future care, communication and decisional satisfaction, and decisional regret in a group of 450 patients aged 65 and over with advanced cancer, as well as analyses of video declarations from 240 of these patients about their wishes. ***Hypothesis****: Patients in the intervention phase (vs. control) will have improved outcomes (communication, decisional satisfaction, decisional regret) and will have better concordance between the content of their video declarations and their documented wishes.*

Table 4. Number of eligible clinics and eligible patients

	# of eligible clinics	# of patients 65+ with Cancer	# of patients 65+ with Advanced Cancer
Duke	12	2890	1341
Mayo	12	3018	701
Northwell	12	4235	2243
Total	36	10143	4285

(The 450 participants that complete the surveys will not be included in the 29,550patients considered for Aim 2 since surveying patients may in and of itself influence the primary and secondary outcomes.)

The patient-centered outcomes will be assessed in a group of 450 patients through surveys. With each clinic contributing survey data only one period before and after intervention, we will use linear mixed models to compare survey responses from intervention and control periods, with clinic as a random effect to take into account of the clustering within clinic. We will include calendar time and any imbalance from patient characteristics in the model to adjust for the potential confounding factors.

The patient surveys allow us to richly characterize the experience of patients and enhance the analysis of the Comprehensive ACP Program. Comprised of multiple previously validated measures as well as two single Likert-scale questions, this survey measures our patient-centered secondary outcomes such as patient confidence that their future medical care will match their values, satisfaction with their clinician's communication, satisfaction with their medical decision, and regret about their medical decision. The first of the single questions, asking patients how confident they are that they will get the type of medical care they want if they become seriously ill, is currently being used in another ACP study by the research team. Although it has not been psychometrically tested, it carries enormous face validity and is the closest approximation we can obtain of goal-concordant care prospectively. The next section is comprised of the 10-item communication subscale from the Consumer Assessment of Health Plans (CAHPS) and asks patients to focus on recent ACP communication with their clinicians and their satisfaction with that communication. This is a well-validated and commonly used measure of clinician communication. The third section of the survey uses the Satisfaction with Decision Scale, a six-item scale that has excellent reliability (Cronbach's alpha = 0.86) and good discriminant validity, to derive patients' satisfaction with their medical decisions in relation to their personal values and goals. This leads directly into the five-item Decision Regret Scale, which measures patients' regret after a decision has been made. This instrument has a Cronbach's alpha of 0.81-0.92 and correlates well with other measures of regret. Finally, the survey asks one question from the End-of-Life Planning survey in the National Health and Aging Trends Study (www.nhats.org) exploring patients' discussions about end-of-life medical treatment with non-clinicians in their lives. The original question asks "have you talked to anyone about the types of medical treatment you want or don't want if you become seriously ill in the future?" and has been amended to say "have you talked with a family member or caregiver about the types of medical treatment you want or don't want if you become seriously ill in the future?" before proceeding to ask with whom that conversation occurred and listing about 20 options. As a single question, we do not believe this has been psychometrically tested and we will have it as an exploratory outcome in this study.

Power Analysis. For our Aim 3 survey outcomes, we will select a group of 450 patients. Assuming an ICC of 0.05, the effective sample size is ~286. The study will have 90% power to detect an effect size of 0.39 and 99% power to detect an effect size of 0.5, which is usually considered as a moderate effect size.

Video declaration. We will conduct a content analysis of the video declarations (N=240). This sample size represents an approach that favors obtaining a wide breadth of data about specific experiences in contrast to more in-depth data that would be obtained by interviewing people at length over several sessions. Our guiding principle will be thematic saturation, such that if we determine that new themes are emerging, we will retain the flexibility to increase the number of people who create video declarations. We will use NVIVO version 11 qualitative software to assist in data management across health systems. To begin, we will remove any inadvertent references to study group, so that Dr. Quintiliani and the RAs will be blinded to. We will transcribe the patient-recorded videos verbatim and add to the transcript any relevant non-verbal information from the video itself, such as expressions of sadness. We will draw from an existing conceptual framework of palliative cancer care to create a preliminary coding framework. The starting coding categories come from our preliminary study of video declarations: 1) ACP; 2) acute issues; 3) psychosocial issues; 4) after death wishes; and, 5) existential and spiritual issues. Dr. Quintiliani will then independently code a subset of 15 videos (5 from each site) adding additional codes as needed to cover emerging topics. Dr. Quintiliani will then show the preliminary coding structure to the entire research team. Codes will be added, refined, and deleted during this process. Once a final coding framework is developed, Dr. Quintiliani and the RAs from each site will each independently code the remaining transcripts, meeting by phone monthly to review progress and resolve any coding discrepancies. To enhance the trustworthiness of the analysis, we will hold at least two peer debriefing meetings with the entire research team to show them the transcripts and the codes applied and ask for their feedback, which will then be incorporated into the coding process. Codes will then be summarized into themes and presented descriptively; illustrative quotations will accompany each category to highlight the content.

In further exploratory analyses, we will evaluate the **clarity** and **comprehensiveness** with which the video declaration communicates preferences. Similarly, we will compare the participant's ACP preferences documented in the video declaration with the ACP preferences documented in the EHR to determine the level of agreement. In this exploratory analysis, we would hypothesize that participants who created their video declaration of preferences after initiation of the intervention would have videos that, (a) have more clarity, and (b) are more consistent with their documented wishes than participants who created their video declaration prior to the initiation of the intervention.

Goal-concordant care. Using a chart abstraction tool we will extract data from the medical record for the last three months of life for all decedent participants who die during the study period regarding: (a) ACP preferences, and (b) care received. Study staff will blind these documents to intervention/control status; this will include the 'shift and truncate' method for obscuring dates while maintaining temporal relationships.(109) We will then independently judge if the care delivered was concordant with a person's wishes as documented at that time. While it is not common in the literature for patients' wishes to change over a short period of time,(110) we will conduct this analysis in a manner that allows for such changes.

For both the clarity and comprehensiveness evaluation and the goal-concordant care assessment we will calculate the extent of agreement using the average *P(e)* between all coder pairs to compute a kappa-like statistic for multiple coders as described by Davies

and Fleiss.(111) Coders will meet to review their determinations and discuss each case for which there was disagreement; final judgements will be determined by consensus.

Dr. Paasche-Orlow at Tufts Medical Center will assist with data analysis but will only receive completely deidentified data and will have no access to identifiers.

Dr. Quintiliani at Tufts Medical Center will assist with data analysis but will only receive completely deidentified data and will have no access to identifiers.

Both Dr. Paasche-Orlow and Dr. Quintiliani will have research staff assisting them with their analysis at Tufts.

SECURING THE DATA

EHR Data Transfer Procedures

1. Representatives from all three health care systems will make semi-annual “dumps”, using appropriately secure tools, of EHR data onto selected servers.
2. Scheduled job run by DFCI will initiate the ingestion of new data as made available.
3. DFCI's system manager replaces person identifiers, including all HIPAA face identifiers, with an alphanumeric ID.

12.0 Provisions to Monitor the Data to Ensure the Safety of Subjects

Data Safety and Monitoring Board (DSMB):

A DSMB has been set up for this large multi-site pragmatic trial. DSMB features will be written in a DSMB charter, including: membership (N=3), meeting schedule, format, and report form, and roles and responsibilities. DSMB members will include a biostatistician and researchers from outside Dana-Farber and MGH with expertise in geriatrics, oncology, ACP, and cluster RCTs. The roles and responsibilities will include, but are not limited to, defining and monitoring the following: adverse events, data quality and handling, recruitment, protocol **modifications or deviations, and all other factors that** might affect study outcome, participant safety, or ethics of the trial. The DSMB will be responsible for making recommendations regarding trial termination or modifications based on these activities. A mechanism for recording and reporting adverse events to the IRB (s) has been developed. We will hold regular conference calls with the DSMB which will provide the opportunity for questions and answers, resolution of issues and reporting. We will also communicate via email.

13. Withdrawal of Subjects

We do not anticipate any circumstances where the patient will withdraw from participation in the study. Study staff will make clear to the patient that participation is entirely voluntary and may be withdrawn at any time and that the decision not to participate will not affect their care, now or in the future.

If a participant chooses to withdraw from the study, or if we terminate a subject's participation in such a research study without regard to the participant's consent, we will retain and analyze already collected data relating to that participant, even if those data include identifiable private information about the participant. We will honor a participant's request to destroy the participant's data or exclude the data from any analysis.

14.0 Risks to Subjects

Potential risks

The interventions are low risk and are the standard of care in many health care systems. The interventions will be available to all patients in the clinic not only patients who are aged 65 or older with advanced cancer. The clinic is the unit of randomization to a minimal risk intervention.

We anticipate little risk to the participants in this study. To be sure, such interactions may make some patients uncomfortable, sad, or even distressed as they contemplate death and dying with their provider. However, such interactions are explicitly part of the standard of care. Patients receiving the intervention will be asked to listen to and watch videos that contain verbal narratives and visual images related to ACP by clinicians in their clinics trained in this practice. The videos describe three levels of care (life-prolonging care, limited care, and comfort care), and/or specific treatments (i.e., CPR, feeding tubes), and therefore may be distressing. Using similar video decision support tools in our prior studies that have included more than three thousand participants, including patients with advanced cancer, over 90% of patients rated the videos as highly acceptable, helpful or extremely helpful, and would recommend or highly recommend them to others. In these prior studies, we have never had to stop an interview because of participant distress. However, as part of clinician training in this trial, clinicians will be instructed that should a patient or family member become distressed while watching a video and prefer not to continue, the video should be stopped.

In addition, there is a risk for loss of privacy in regards to the video declaration, but we will have a series of mutually reinforcing measures to mitigate this risk.

Protections against risk

We will emphasize that participation in this research is voluntary.

Qualitative interviews with providers, staff, and management will be conducted by personnel who have no affiliation to these subjects' employer. It is quite important that providers, staff, and management are able to share openly, even if their feedback may be critical.

We will transmit no identifiable data back to the clinic sites to avoid any risk of retribution, retaliation, or adverse consequence to disclosures which may come about through this process.

15.0 Potential Benefits to Subjects

Potential Benefits of the Proposed Research to Human Subjects and Others

There is the potential for patients and clinicians in the clinics to benefit from the study by having their treatments better aligned with their preferences. The minor risks for the participants in this study may be considered counterbalanced by the potential direct benefits and knowledge gained. The results gleaned from the study are intended to improve the ACP of the overall outpatient clinic population, and particularly those with advanced cancer. Thus, the risk/benefit balance for this study appears favorable.

Importance of the Knowledge to be Gained

There is the potential to validate an intervention that could ensure that treatments are better aligned with patients' preferences. The minor risks for the participants in this study may be considered counterbalanced by the potential direct benefits and knowledge gained. The results gleaned from the study are intended to improve the ACP of the overall outpatient clinic population, and particularly those with advanced cancer. Thus, the risk/benefit balance for this study appears favorable.

16.0 Vulnerable Populations

Our project does not involve any individuals from vulnerable populations.

17.0 Community-Based Participatory Research

N/A

18.0 Sharing of Results with Patients

The results will be shared via a patient friendly pamphlet that explains the results of the study and is disseminated in participating clinics. An e-version will also be available on any websites affiliated with the practice.

19.0 Setting

Locations:
We will recruit patients from 39 oncology clinics served by three major health care systems: Duke Health (North Carolina), the Mayo Clinic (Minnesota), and Northwell Health (New York).

Health Care System Partners:

Three health care systems are participating in the study, each contributing 13 oncology clinics: Duke Health, Mayo Clinic, and Northwell.

Clinic Eligibility:

Clinic eligibility criteria include:

- More than one oncologist
- Serve a patient population that is at least 30% age of 65 or older

Eligibility Estimates

We anticipated that a total of 13 clinic sites per health care system would be eligible for the trial. For the UG3 year, the pilot was selected, leaving 12 clinics for random assignment during the UH3 year.

Identification of Eligible Clinics

Candidate clinics were identified by the research team at each health care system site using EHR data to establish patient mix (i.e., >30% age of 65 or older).

The research team at each site along with leaders at the health care system partners reviewed these lists for:

- Recent turnover in Administrator or Director of the Clinic
- Current new initiatives/competing demands

Each site then returned the list of clinics to the study statistician who will then randomize the sites.

Clinic Randomization

- The randomization scheme for the 36 participating clinics at the three health care systems will be finalized during the UG3 year.

- During the first year of the UH3 phase and continuing every six months (a total of six steps), we will randomize 2 oncology clinics in each of our three health care systems to receiving the intervention. Prior to randomization, the size of participant clinics will be determined based on the number of oncologists on staff (≤ 3 vs. >3). To balance the size of clinics between intervention and control

phases of the trial, we will pair clinics of different sizes (one with ≤ 3 and one with >3) to receive intervention at the same time.

Masking/Blinding

Various levels of blinding will be used in this trial.

- Research site-PIs (Drs. Pollak, Zafar, Tilburt, Loprinzi, Martins-Welch and Carney) at each of our sites will know the randomization order and which clinics have received the intervention and when.
- Selected members of the data management (Drs. Lakin and Lindvall, and Dan Gundersen) and implementation teams (Drs. Paasche-Orlow, Barry and Volandes) are unblinded to all clinic assignments and outcomes as a function of their roles in working with the data and generating monthly video adherence reports for the clinics using the intervention.
- Drs. Tulsky will be aware of the identity of the intervention clinics, but will remain blinded to all outcomes.
- All other project staff not involved with data management and implementation will be blinded to clinic assignments and outcomes.

Each clinic has an **advisory board,** who will be involved in the project throughout the study.

Each health care system has a **regulatory board**, which will use DFCI's OHRS approval as their main regulatory agent. Each of the 3 sites, Duke, Mayo Clinic and Northwell Health, have conceded to allow that the DFCI IRB will be the IRB of record. The sites will be following rules and regulations set forth by DFCI OHRS.

20.0 Resources Available

James A. Tulsky, MD, Co-**Principal Investigator (2.4 CM, Years 1-5):** Dr. Tulsky is Professor of Medicine at Harvard Medical School and Chair, Department of Psychosocial Oncology and Palliative Care at DFCI. He has led multiple federally funded studies examining patient-provider communication, and is a Founding Director of VitalTalk, a non-profit organization devoted to teaching physicians communication skills (www.VitalTalk.org). Dr. Tulsky will provide scientific leadership over all aspects of the study. He will oversee the conduct of the study including protocol development, training of research staff, codebook development, data analysis and manuscript writing.

Angelo Volandes, MD, MPH, Co-Principal Investigator. Dr. Volandes is a general internist and clinical researcher with past training in medicine, decision-making, bioethics, and film-making. His research career has been dedicated to developing and evaluating video decision support tools for life-limiting conditions. He currently leads an innovative team of video decision scientists at the Massachusetts General Hospital. He

has successfully led many studies funded by the NIH evaluating the efficacy of these tools, the results of which have been published in top-tier journals.

Feasibility:

- **All investigators, research assistants, and study staff will be oriented to the protocol during an in-person meeting.** During this meeting, the PIs and PM will provide a detailed orientation to all study procedures and all study staff members' duties and functions. Each site will also have electronic and hard copies of the project manual and protocol which will be made available to study staff. Regular check-in conference calls with study staff will be used to provide updates on the protocol if changes have been made, discuss issues at the individual sites with regard to recruitment, administration of the intervention, assessment, or any other issue that may arise. All persons assisting with the research will undergo training on the protocol including training on informed consent.

- The study will leverage the state-of-the-art EHRs of three large health care systems: We will establish a unique, rigorous data infrastructure to identify target patient groups (older patients with advanced cancer) in a timely manner. The co-PIs have a long history of prior work with the three health care systems, and have published with their co-Investigators. ***The proposed work will leverage this exceptional field experience and infrastructure, and supports the feasibility of the study.***

21.0 Prior Approvals

N/A

22.0 Recruitment Methods

There will be no local subjects recruited for this project.

23.0 Local Number of Subjects

There will be no local subjects recruited for this project.

24.0 Provisions to Protect the Privacy Interests of Subjects

The risks in this study are minimal and non-medical in nature. The primary risk is loss of confidentiality. To minimize the likelihood of a breach, we will collect only electronic data and anonymous data when possible. Only the minimum amount of PHI necessary will be collected from study participants, including oncologists and patients, and all data will be transmitted via secure, institutionally approved methods. We will emphasize that participation in this research is voluntary.

Qualitative interviews with providers, staff, and management will be conducted by personnel who have no affiliation to these subjects' employer. It is quite important that providers, staff, and management will be able to share openly, even if their feedback may be critical.

We will transmit no identifiable data back to the clinic sites to avoid any risk of retribution, retaliation, or adverse consequence to disclosures which may come about through this process.

Oversight and Protocol Compliance: All research personnel will participate in human subjects training, an annual booster training, one-time data integrity and security training, and training on the protocol, which will include simulation and practice for all protocol procedures including obtaining informed consent and confirming comprehension. We will have regular team meetings and regular surveying reliability checks to ensure protocol compliance. Patient surveyors will be supervised through monthly conference calls and one-on-one communication, as needed. The procedures described here address our efforts to minimize the risk of breach of confidentiality and to ensure that procedures remain in place for the protection of human subjects. All data management will be conducted at DFCI. The Center's researchers and staff have many years of experience working with similar data files.

Adverse Event Reporting: This study presents minimal risk to participants, and there are no adverse events given that ACP is part of the standard of care. Nonetheless, the PIs will monitor and report unforeseen adverse events to the DFCI IRB. If there are any concerns about privacy, will be refer the patient to the DFCI Privacy Officer, and we will refer any significant complaints to the DFCI Office of Research Studies.

As this is a palliative care study, it is not unexpected for participants to pass away during the course of the research. Thus, a death would not constitute an adverse event and so patient deaths will not be reported as AEs.

25.0 Compensation for Research-Related Injury

N/A

26.0 Economic Burden to Subjects

Subjects will not be responsible for any costs relating to participation in this research.

27.0 Consent Process

Non-English Speaking Subjects = N/A

Subjects who are not yet adults (infants, children, teenagers) = N/A

Cognitively Impaired Adults = N/A

Adults Unable to Consent = N/A

1.DATA OBTAINED FROM THE EHR:
To obtain personal health information from the EHRs, a waiver of HIPAA authorization will be sought.

RECRUITING THE PATIENTS WHO WILL COMPLETE THE SURVEYS:
For those patients meeting all the criteria for advanced cancer, the RA will randomize the order of scheduled patients and then start at the top of this list and go down until fulfilling the enrollment target for the survey. The RA will then contact the patient's primary oncologist by email to solicit his/her opinion as to whether the patient is otherwise appropriate to approach for participation based on the clinician's more intimate knowledge of the patient's clinical status, psychological disposition, and decision-making capacity. Once a potential participant is identified, a letter outlining the project will be mailed or emailed using the institution's secured email system to the patient prior to the clinic visit.

2. PATIENT SUB-GROUP WHO WILL COMPLETE THE SURVEY:
For our group of patients (N=450) being surveyed for patient-centered secondary outcomes, verbal informed consent will be obtained for the verbal survey.

3. PATIENT SUB-GROUP WHO WILL COMPLETE THE VIDEO DECLARATION:

For those participants who we see in person, we will obtain signed informed consent following DF/HCC CON-100 policy. For those participants who we are unable to see in person/ are unwilling to come to clinic, we request a waiver of documentation of consent as this protocol presents no more than minimal risk and only involves procedures of which consent is not required outside of a research context. While participants will be presented with all required elements of consent via a detailed information sheet that is (emailed, described over the phone etc), a signed document will not be obtained. DFCI will host these ViDecs via HIPAA compliant ZOOM, while external sites will host their ViDecs via a HIPAA compliant software of their choice: Duke will use ZOOM, Mayo will use ZOOM and Northwell will use Microsoft Teams. Participants may also type out their answers to the questions in an email or provide them over the phone while the RA transcribes their answers.

We are offering sites the option of calling selected potential ACP PEACE survey participants after they have received the recruitment letter from their physicians, yet before study staff meet them in-person at the clinic to obtain written informed consent. These follow-up phone calls allow research staff to introduce participants to the concept of video declaration prior to the in-person meeting. We hope this will allow patient

participants more time to consider this request since video declaration will be novel to most patients. We hope that providing this extra time will allow them more reflection rather than being asked to decide "on the spot" during the clinic visit.

For sites that choose to make these pre-visit phone calls, we will include a patient opt-out option in the body of the "recruitment letter from the physician to the patient" and a prepaid, addressed postcard. They can either send this postcard to the study staff or call a number printed on the card. In either case, if we hear from the patient, we will make no more contact. If we don't receive an opt-out call or returned postcard, then after 21 days our RA will call the patient to invite them to join the study.

On the day of his/her clinic visit, the patient will be approached at the conclusion of the scheduled clinic appointment to further explain the study and obtain verbal informed consent for the verbal survey. The RA will verify the ability of the participant to provide consent by explaining the nature of the study and having the participant repeat (teach-back) the aims and risks of the study. Only those participants who can understand the aims of the project, what their involvement entails, that participation is voluntary, and the risks and benefits of participation will be eligible for the study.

28. Subject Registration Procedures

1. Institutions will register eligible participants in the Clinical Trials Management System (CTMS) OnCore as required by DF/HCC Policy REGIST-101. Summary accrual reporting will be utilized, and accrual data will be entered into OnCore at least monthly while the protocol is open to accrual.

2. The coordinating center will produce site specific accrual reports and update the summary accrual report immediately after data transfer occurrence. Data from each site are transmitted to Daniel Gundersen (DanielA_Gundersen@dfci.harvard.edu) who will produce the site accrual data and update the summary accrual report.

29. Process to Document Consent in Writing

We will be following DF/HCC Policy CON-100: Informed Consent Process.

The submitted DFCI consent will serve as a master template consent, and the subjects will be consented locally, with consents approved by their local IRBs.

30. Drugs or Devices

N/A

APPENDIX A

In order to maximize the learning of this study and to make sure that the intervention is replicable as much as possible, we have described in this Appendix our implementation strategy.

1) **Name it.** The overall intervention is called the Comprehensive Advance Care Planning (ACP) Program. The Comprehensive ACP Program has two components:

a) A clinician-facing component, VitalTalk Training to improve communication skills about advance care planning for key clinic personnel
b) A patient-facing component, Viewing of ACP Videos as an adjunct to ACP discussions between clinic staff and eligible patients in the practice.

2) **Define it**. For the clinician intervention, the VitalTalk program is the most widely disseminated teaching method that focuses on patient-centered communication skills training. Thus, this part of the intervention is best considered a clinician training intervention. For patients, video decision aids to better educate and inform decision making are commonly used, and the ACP Decisions video-based tools are the best example of these. These videos attempt to overcome literacy barriers and to present potential scenarios with a sense of reality lacking in verbal descriptions. This part of the intervention is best considered a patient decision support intervention aimed at insuring patients are informed and involved in their ACP planning decisions. Prior work, including several RCTs, supports the efficacy of the ACP Decisions videos and the VitalTalk program in patients with advanced cancer. To improve the intensity of the patient-facing component, there will also be an audit and feedback component focused on the intensity of viewing if the ACP videos by eligible patients. These trainings may be done in-person or virtually.
3) **Operationalize It.**

a) The actors
For the VitalTalk Training the actors are the project's research staff who will visit each participating site to train the clinicians. For the Viewing ACP Decisions Videos, research staff will also play a role training clinic personnel in the use of the ACP Videos to support ACP conversations. However, the main actors will be clinic personnel who will provide access and encouragement for ACP video viewing, and then "close the loop" by arranging ACP conversations between clinicians and patients. The audit and feedback component will be performed by the project's research staff.

b) The action

For VitalTalk, the action is the in-person or virtual training for clinicians in each intervention practice. For Viewing of ACP Videos, the action begins with training the practice's clinic team in the use of ACP Videos in conjunction with each site's VitalTalk training. The ultimate action is clinic personnel prescribing ACP Videos ahead of eligible patient visits to the practice, and face-to-face viewing of the ACP Videos on tablets in the practice in conjunction with ACP Conversations. The audit and feedback component will be provision of reports on intensity of use of the ACP Videos among eligible patients in the practice at the end of each intervention cycle as well as ACP completion.

c) Action target

Training in the skills of having ACP conversations and prescribing ACP planning videos should help reduce the barriers to initiating ACP discussions in these oncology practices. As most patients welcome such discussions and respond very positively to the ACP Videos, these patient reactions should provide an ongoing feedback loop to reinforce the implementation of the intervention over time. Providing regular feedback on the intensity of use of the ACP Videos will provide another reinforcement to their use.

d) Temporality

ACP conversations with eligible patients should begin immediately after the training, as eligible patients are seen in the practice. Similarly, ACP Videos should be viewed with practice personnel in the office immediately after the training. If desired, clinic staff or research staff can send patients links to the relevant videos by mail or secured email with a recommendation to review them prior to an upcoming visit. However, the loop should be closed with an ACP discussion at the upcoming visit. Feedback on ACP Video use will be provided to each practice at the end of each 6-month cycle after implementation.

e) Dose

The dose of VitalTalk training is uniform across the participating practices, with one daylong training session conducted at the beginning of each site's implementation period. However, the dose of ACP Video viewing will vary by practice. The intensity of use of the videos will be monitored by making a ratio of the number of videos viewed overall and separately inside or outside of office visits (which the ACP Decisions sire tracks separately) to the number of eligible patients in the practice during each 6-month intervention cycle.

f) The implementation outcome affected

The primary outcome of the study is the documentation of ACP conversations in the electronic medical record. The main intermediate implementation outcome will be the intensity of ACP Video viewing, as measured above.

g) The justification

This strategy represents the first Comprehensive ACP Program combining two well-tested, evidence-based, and complementary interventions, that treat patients and clinicians as equal stakeholders by providing both with the communication skills and tools needed to optimally engage with ACP. A modified conceptual model integrating patient-professional communication such as ACP conversations has been developed by Feldman-Stewart and colleagues. The potential for a Comprehensive ACP Program to improve outcomes based on this model provides the theoretical framework for the trial. The 4 elements of the model are: 1. interventions for patients (ACP Videos) and clinicians (VitalTalk); 2. moderators for patients (demographics, cancer type/stage, functional status) and clinicians (demographics, experience); 3. mediators for patients (self-efficacy, knowledge) and clinicians (inclination toward social and emotional aspects of patient care); and, 4. ACP outcomes.

REFERENCES:

1. Teno JM, Clarridge BR, Casey V, Welch LC, Wetle T, Shield R, Mor V. Family perspectives on end-of-life care at the last place of care. JAMA : the journal of the American Medical Association. 2004;291(1):88-93. Epub 2004/01/08. doi: 10.1001/jama.291.1.88. PubMed PMID: 14709580.
2. Teno JM, Gozalo PL, Bynum JP, Leland NE, Miller SC, Morden NE, Scupp T, Goodman DC, Mor V. Change in end-of-life care for Medicare beneficiaries: site of death, place of care, and health care transitions in 2000, 2005, and 2009. JAMA : the journal of the American Medical Association. 2013;309(5):470-7. Epub 2013/02/07. doi: 10.1001/jama.2012.207624. PubMed PMID: 23385273; PMCID: Pmc3674823.
3. A controlled trial to improve care for seriously ill hospitalized patients. The study to understand prognoses and preferences for outcomes and risks of treatments (SUPPORT). The SUPPORT Principal Investigators. JAMA : the journal of the American Medical Association. 1995;274(20):1591-8. Epub 1995/11/22. PubMed PMID: 7474243.
4. Smedira NG, Evans BH, Grais LS, Cohen NH, Lo B, Cooke M, Schecter WP, Fink C, Epstein-Jaffe E, May C, et al. Withholding and withdrawal of life support from the critically ill. The New England journal of medicine. 1990;322(5):309-15. Epub 1990/02/01. doi: 10.1056/nejm199002013220506. PubMed PMID: 2296273.
5. Earle CC, Neville BA, Landrum MB, Ayanian JZ, Block SD, Weeks JC. Trends in the aggressiveness of cancer care near the end of life. Journal of clinical oncology : official journal of the American Society of Clinical Oncology. 2004;22(2):315-21. Epub 2004/01/15. doi: 10.1200/jco.2004.08.136. PubMed PMID: 14722041.
6. Earle CC, Landrum MB, Souza JM, Neville BA, Weeks JC, Ayanian JZ. Aggressiveness of cancer care near the end of life: is it a quality-of-care issue? Journal of clinical oncology : official journal of the American Society of Clinical Oncology. 2008;26(23):3860-6. Epub 2008/08/09. doi: 10.1200/jco.2007.15.8253. PubMed PMID: 18688053; PMCID: PMC2654813.
7. Ahronheim JC, Morrison RS, Baskin SA, Morris J, Meier DE. Treatment of the dying in the acute care hospital. Advanced dementia and metastatic cancer. Archives of internal medicine. 1996;156(18):2094-100. Epub 1996/10/14. PubMed PMID: 8862102.
8. NIH State-of-the-Science Conference Statement on improving end-of-life care. NIH consensus and state-of-the-science statements. 2004;21(3):1-26. Epub 2007/02/20. PubMed PMID: 17308546.
9. Institute of Medicine. Dying in America: Improving quality and honoring individual preferences near the end of life. Washington, D.C.2014.
10. Tulsky JA. Improving quality of care for serious illness: findings and recommendations of the Institute of Medicine report on dying in America. JAMA internal medicine. 2015;175(5):840-1. Epub 2015/03/03. doi: 10.1001/jamainternmed.2014.8425. PubMed PMID: 25730201.

11. Aitken PV, Jr. Incorporating advance care planning into family practice [see comment]. American family physician. 1999;59(3):605-14, 17-20. Epub 1999/02/25. PubMed PMID: 10029787.
12. Emanuel LL, von Gunten CF, Ferris FD. Advance care planning. Archives of family medicine. 2000;9(10):1181-7. Epub 2000/12/15. PubMed PMID: 11115227.
13. Emanuel LL, Barry MJ, Stoeckle JD, Ettelson LM, Emanuel EJ. Advance directives for medical care--a case for greater use. The New England journal of medicine. 1991;324(13):889-95. Epub 1991/03/28. doi: 10.1056/nejm199103283241305. PubMed PMID: 2000111.
14. Sudore RL, Fried TR. Redefining the "planning" in advance care planning: preparing for end-of-life decision making. Annals of internal medicine. 2010;153(4):256-61. Epub 2010/08/18. doi: 10.7326/0003-4819-153-4-201008170-00008. PubMed PMID: 20713793; PMCID: Pmc2935810.
15. Tulsky JA BM, Butow PN, Hickman SE, Mack JW, Morrison RS, Street RL, Sudore RL, White DB, Pollak KI. A research agenda for communication in serious illness. JAMA Intern Med 2017; in press.
16. Mack JW, Weeks JC, Wright AA, Block SD, Prigerson HG. End-of-life discussions, goal attainment, and distress at the end of life: predictors and outcomes of receipt of care consistent with preferences. Journal of clinical oncology : official journal of the American Society of Clinical Oncology. 2010;28(7):1203-8. Epub 2010/02/04. doi: 10.1200/jco.2009.25.4672. PubMed PMID: 20124172; PMCID: Pmc2834470.
17. Wright AA, Zhang B, Ray A, Mack JW, Trice E, Balboni T, Mitchell SL, Jackson VA, Block SD, Maciejewski PK, Prigerson HG. Associations between end-of-life discussions, patient mental health, medical care near death, and caregiver bereavement adjustment. JAMA : the journal of the American Medical Association. 2008;300(14):1665-73. Epub 2008/10/09. doi: 10.1001/jama.300.14.1665. PubMed PMID: 18840840; PMCID: Pmc2853806.
18. Detering KM, Hancock AD, Reade MC, Silvester W. The impact of advance care planning on end of life care in elderly patients: randomised controlled trial. BMJ (Clinical research ed). 2010;340:c1345. Epub 2010/03/25. doi: 10.1136/bmj.c1345. PubMed PMID: 20332506; PMCID: Pmc2844949.
19. El-Jawahri A, Podgurski LM, Eichler AF, Plotkin SR, Temel JS, Mitchell SL, Chang Y, Barry MJ, Volandes AE. Use of video to facilitate end-of-life discussions with patients with cancer: a randomized controlled trial. Journal of clinical oncology : official journal of the American Society of Clinical Oncology. 2010;28(2):305-10. Epub 2009/12/02. doi: 10.1200/jco.2009.24.7502. PubMed PMID: 19949010; PMCID: PMC3040012.
20. Volandes AE, Paasche-Orlow MK, Mitchell SL, El-Jawahri A, Davis AD, Barry MJ, Hartshorn KL, Jackson VA, Gillick MR, Walker-Corkery ES, Chang Y, Lopez L, Kemeny M, Bulone L, Mann E, Misra S, Peachey M, Abbo ED, Eichler AF, Epstein AS, Noy A, Levin TT, Temel JS. Randomized controlled trial of a video decision support tool for cardiopulmonary resuscitation decision making in advanced cancer. Journal of clinical oncology : official journal of the American

Society of Clinical Oncology. 2013;31(3):380-6. Epub 2012/12/13. doi: 10.1200/jco.2012.43.9570. PubMed PMID: 23233708; PMCID: PMC4090424.

21. El-Jawahri A, Mitchell SL, Paasche-Orlow MK, Temel JS, Jackson VA, Rutledge RR, Parikh M, Davis AD, Gillick MR, Barry MJ, Lopez L, Walker-Corkery ES, Chang Y, Finn K, Coley C, Volandes AE. A Randomized Controlled Trial of a CPR and Intubation Video Decision Support Tool for Hospitalized Patients. Journal of general internal medicine. 2015;30(8):1071-80. Epub 2015/02/19. doi: 10.1007/s11606-015-3200-2. PubMed PMID: 25691237; PMCID: PMC4510229.
22. Back AL, Arnold RM, Baile WF, Fryer-Edwards KA, Alexander SC, Barley GE, Gooley TA, Tulsky JA. Efficacy of communication skills training for giving bad news and discussing transitions to palliative care. Archives of internal medicine. 2007;167(5):453-60. Epub 2007/03/14. doi: 10.1001/archinte.167.5.453. PubMed PMID: 17353492.
23. Tulsky JA, Arnold RM, Alexander SC, Olsen MK, Jeffreys AS, Rodriguez KL, Skinner CS, Farrell D, Abernethy AP, Pollak KI. Enhancing communication between oncologists and patients with a computer-based training program: a randomized trial. Annals of internal medicine. 2011;155(9):593-601. Epub 2011/11/02. doi: 10.7326/0003-4819-155-9-201111010-00007. PubMed PMID: 22041948; PMCID: PMC3368370.
24. Berger NA, Savvides P, Koroukian SM, Kahana EF, Deimling GT, Rose JH, Bowman KF, Miller RH. Cancer in the elderly. Transactions of the American Clinical and Climatological Association. 2006;117:147-55; discussion 55-6. Epub 2008/06/06. PubMed PMID: 18528470; PMCID: PMC1500929.
25. Howlader N, Noone AM, Krapcho M, Miller D, Bishop K, Altekruse SF, Kosary CL, Yu M, Ruhl J, Tatalovich Z, Mariotto A, Lewis DR, Chen HS, Feuer EJ, Cronin KA (eds). SEER Cancer Statistics Review, 1975-2013, National Cancer Institute. Bethesda, MD, https://seer.cancer.gov/csr/1975_2013/, based on November 2015 SEER data submission, posted to the SEER web site, April 2016.
26. Yabroff KR, Lamont EB, Mariotto A, Warren JL, Topor M, Meekins A, Brown ML. Cost of care for elderly cancer patients in the United States. Journal of the National Cancer Institute. 2008;100(9):630-41. Epub 2008/05/01. doi: 10.1093/jnci/djn103. PubMed PMID: 18445825.
27. U.S. Department of Health and Human Services; Office of the Assistant Secretary for 2. Planning and Evaluation; Office of Disability, Aging and Long-Term Care Policy. Advance directives and advance care planning: Report to Congress. 2008. http://aspe.hhs.gov/daltcp/reports/2008/ADCongRpt.pdf. Accessed May 25, 2017.
28. Mitchell SL, Kiely DK, Hamel MB. Dying with advanced dementia in the nursing home. Archives of internal medicine. 2004;164(3):321-6. Epub 2004/02/11. doi: 10.1001/archinte.164.3.321. PubMed PMID: 14769629.
29. Mitchell SL, Teno JM, Kiely DK, Shaffer ML, Jones RN, Prigerson HG, Volicer L, Givens JL, Hamel MB. The clinical course of advanced dementia. The New England journal of medicine. 2009;361(16):1529-38. Epub 2009/10/16. doi: 10.1056/NEJMoa0902234. PubMed PMID: 19828530; PMCID: Pmc2778850.

30. Bernacki RE, Block SD. Communication About Serious Illness Care Goals: A Review and Synthesis of Best Practices. JAMA internal medicine. 2014. Epub 2014/10/21. doi: 10.1001/jamainternmed.2014.5271. PubMed PMID: 25330167.
31. Thorne SE, Bultz BD, Baile WF. Is there a cost to poor communication in cancer care?: a critical review of the literature. Psycho-oncology. 2005;14(10):875-84; discussion 85-6. Epub 2005/10/04. doi: 10.1002/pon.947. PubMed PMID: 16200515.
32. Heyland DK, Barwich D, Pichora D, Dodek P, Lamontagne F, You JJ, Tayler C, Porterfield P, Sinuff T, Simon J. Failure to engage hospitalized elderly patients and their families in advance care planning. JAMA internal medicine. 2013;173(9):778-87. Epub 2013/04/03. doi: 10.1001/jamainternmed.2013.180. PubMed PMID: 23545563.
33. Teno JM, Branco KJ, Mor V, Phillips CD, Hawes C, Morris J, Fries BE. Changes in advance care planning in nursing homes before and after the patient Self-Determination Act: report of a 10-state survey. Journal of the American Geriatrics Society. 1997;45(8):939-44. Epub 1997/08/01. PubMed PMID: 9256845.
34. Mitchell SL, Teno JM, Intrator O, Feng Z, Mor V. Decisions to forgo hospitalization in advanced dementia: a nationwide study. Journal of the American Geriatrics Society. 2007;55(3):432-8. Epub 2007/03/08. doi: 10.1111/j.1532-5415.2007.01086.x. PubMed PMID: 17341248.
35. White DB, Arnold RM. The evolution of advance directives. JAMA : the journal of the American Medical Association. 2011;306(13):1485-6. Epub 2011/10/06. doi: 10.1001/jama.2011.1430. PubMed PMID: 21972313.
36. Mystakidou K, Parpa E, Tsilila E, Katsouda E, Vlahos L. Cancer information disclosure in different cultural contexts. Supportive care in cancer : official journal of the Multinational Association of Supportive Care in Cancer. 2004;12(3):147-54. Epub 2004/04/13. doi: 10.1007/s00520-003-0552-7. PubMed PMID: 15074312.
37. Johnson KS, Kuchibhatla M, Tulsky JA. What explains racial differences in the use of advance directives and attitudes toward hospice care? Journal of the American Geriatrics Society. 2008;56(10):1953-8. Epub 2008/09/06. doi: 10.1111/j.1532-5415.2008.01919.x. PubMed PMID: 18771455; PMCID: PMC2631440.
38. Mack JW, Paulk ME, Viswanath K, Prigerson HG. Racial disparities in the outcomes of communication on medical care received near death. Archives of internal medicine. 2010;170(17):1533-40. Epub 2010/09/30. doi: 10.1001/archinternmed.2010.322. PubMed PMID: 20876403; PMCID: PMC3739279.
39. Smith AK, McCarthy EP, Paulk E, Balboni TA, Maciejewski PK, Block SD, Prigerson HG. Racial and ethnic differences in advance care planning among patients with cancer: impact of terminal illness acknowledgment, religiousness, and treatment preferences. Journal of clinical oncology : official journal of the American Society of Clinical Oncology. 2008;26(25):4131-7. Epub 2008/09/02. doi: 10.1200/jco.2007.14.8452. PubMed PMID: 18757326; PMCID: PMC2654372.

40. Morrison RS, Meier DE. High rates of advance care planning in New York City's elderly population. Archives of internal medicine. 2004;164(22):2421-6. Epub 2004/12/15. doi: 10.1001/archinte.164.22.2421. PubMed PMID: 15596631.
41. Fagerlin A, Schneider CE. Enough. The failure of the living will. The Hastings Center report. 2004;34(2):30-42. Epub 2004/05/26. PubMed PMID: 15156835.
42. Loewenstein G. Hot-cold empathy gaps and medical decision making. Health psychology : official journal of the Division of Health Psychology, American Psychological Association. 2005;24(4s):S49-56. Epub 2005/07/28. doi: 10.1037/0278-6133.24.4.s49. PubMed PMID: 16045419.
43. Billings JA. The need for safeguards in advance care planning. Journal of general internal medicine. 2012;27(5):595-600. Epub 2012/01/13. doi: 10.1007/s11606-011-1976-2. PubMed PMID: 22237664; PMCID: Pmc3326115.
44. Billings JA, Bernacki R. Strategic targeting of advance care planning interventions: the Goldilocks phenomenon. JAMA internal medicine. 2014;174(4):620-4. Epub 2014/02/05. doi: 10.1001/jamainternmed.2013.14384. PubMed PMID: 24493203.
45. Lakin JR, Block SD, Billings JA, Koritsanszky LA, Cunningham R, Wichmann L, Harvey D, Lamey J, Bernacki RE. Improving Communication About Serious Illness in Primary Care: A Review. JAMA internal medicine. 2016. Epub 2016/07/12. doi: 10.1001/jamainternmed.2016.3212. PubMed PMID: 27398990.
46. Winter L, Parks SM, Diamond JJ. Ask a different question, get a different answer: why living wills are poor guides to care preferences at the end of life. Journal of palliative medicine. 2010;13(5):567-72. Epub 2010/04/10. doi: 10.1089/jpm.2009.0311. PubMed PMID: 20377499; PMCID: PMC2938890.
47. Patel K, Janssen DJ, Curtis JR. Advance care planning in COPD. Respirology (Carlton, Vic). 2012;17(1):72-8. Epub 2011/10/20. doi: 10.1111/j.1440-1843.2011.02087.x. PubMed PMID: 22008225.
48. Davison SN, Simpson C. Hope and advance care planning in patients with end stage renal disease: qualitative interview study. BMJ (Clinical research ed). 2006;333(7574):886. Epub 2006/09/23. doi: 10.1136/bmj.38965.626250.55. PubMed PMID: 16990294; PMCID: Pmc1626305.
49. Quill TE. Perspectives on care at the close of life. Initiating end-of-life discussions with seriously ill patients: addressing the "elephant in the room". JAMA : the journal of the American Medical Association. 2000;284(19):2502-7. Epub 2000/11/14. PubMed PMID: 11074781.
50. Sharman SJ, Garry M, Jacobsen JA, Loftus EF, Ditto PH. False memories for end-of-life decisions. Health psychology : official journal of the Division of Health Psychology, American Psychological Association. 2008;27(2):291-6. Epub 2008/04/02. doi: 10.1037/0278-6133.27.2.291. PubMed PMID: 18377150.
51. Fischer GS, Tulsky JA, Rose MR, Siminoff LA, Arnold RM. Patient knowledge and physician predictions of treatment preferences after discussion of advance directives. Journal of general internal medicine. 1998;13(7):447-54. Epub 1998/08/01. PubMed PMID: 9686710; PMCID: PMC1496983.
52. De Vleminck A, Houttekier D, Pardon K, Deschepper R, Van Audenhove C, Vander Stichele R, Deliens L. Barriers and facilitators for general practitioners to

engage in advance care planning: a systematic review. Scandinavian journal of primary health care. 2013;31(4):215-26. Epub 2013/12/05. doi: 10.3109/02813432.2013.854590. PubMed PMID: 24299046; PMCID: Pmc3860298.

53. De Vleminck A, Pardon K, Beernaert K, Deschepper R, Houttekier D, Van Audenhove C, Deliens L, Vander Stichele R. Barriers to advance care planning in cancer, heart failure and dementia patients: a focus group study on general practitioners' views and experiences. PloS one. 2014;9(1):e84905. Epub 2014/01/28. doi: 10.1371/journal.pone.0084905. PubMed PMID: 24465450; PMCID: Pmc3897376.
54. Ahluwalia SC, Bekelman DB, Huynh AK, Prendergast TJ, Shreve S, Lorenz KA. Barriers and Strategies to an Iterative Model of Advance Care Planning Communication. The American journal of hospice & palliative care. 2014. Epub 2014/07/06. doi: 10.1177/1049909114541513. PubMed PMID: 24988894.
55. Evans LR, Boyd EA, Malvar G, Apatira L, Luce JM, Lo B, White DB. Surrogate decision-makers' perspectives on discussing prognosis in the face of uncertainty. American journal of respiratory and critical care medicine. 2009;179(1):48-53. Epub 2008/10/22. doi: 10.1164/rccm.200806-969OC. PubMed PMID: 18931332; PMCID: Pmc2615661.
56. Fried TR, O'Leary J, Van Ness P, Fraenkel L. Inconsistency over time in the preferences of older persons with advanced illness for life-sustaining treatment. Journal of the American Geriatrics Society. 2007;55(7):1007-14. Epub 2007/07/05. doi: 10.1111/j.1532-5415.2007.01232.x. PubMed PMID: 17608872; PMCID: Pmc1948955.
57. Keating NL, Landrum MB, Rogers SO, Jr., Baum SK, Virnig BA, Huskamp HA, Earle CC, Kahn KL. Physician factors associated with discussions about end-of-life care. Cancer. 2010;116(4):998-1006. Epub 2010/01/13. doi: 10.1002/cncr.24761. PubMed PMID: 20066693; PMCID: PMC2819541.
58. Morrison RS, Morrison EW, Glickman DF. Physician reluctance to discuss advance directives. An empiric investigation of potential barriers. Archives of internal medicine. 1994;154(20):2311-8. Epub 1994/10/24. PubMed PMID: 7944853.
59. Smith AK, Williams BA, Lo B. Discussing overall prognosis with the very elderly. The New England journal of medicine. 2011;365(23):2149-51. Epub 2011/12/14. doi: 10.1056/NEJMp1109990. PubMed PMID: 22150033; PMCID: Pmc3760676.
60. Zier LS, Sottile PD, Hong SY, Weissfield LA, White DB. Surrogate decision makers' interpretation of prognostic information: a mixed-methods study. Annals of internal medicine. 2012;156(5):360-6. Epub 2012/03/07. doi: 10.7326/0003-4819-156-5-201203060-00008. PubMed PMID: 22393131; PMCID: PMC3530840.
61. Shalowitz DI, Garrett-Mayer E, Wendler D. The accuracy of surrogate decision makers: a systematic review. Archives of internal medicine. 2006;166(5):493-7. Epub 2006/03/15. doi: 10.1001/archinte.166.5.493. PubMed PMID: 16534034.

62. Sudore RL, Schillinger D, Knight SJ, Fried TR. Uncertainty about advance care planning treatment preferences among diverse older adults. Journal of health communication. 2010;15 Suppl 2:159-71. Epub 2010/09/29. doi: 10.1080/10810730.2010.499982. PubMed PMID: 20845201.
63. Givens JL, Kiely DK, Carey K, Mitchell SL. Healthcare proxies of nursing home residents with advanced dementia: decisions they confront and their satisfaction with decision-making. Journal of the American Geriatrics Society. 2009;57(7):1149-55. Epub 2009/06/03. doi: 10.1111/j.1532-5415.2009.02304.x. PubMed PMID: 19486200; PMCID: PMC2796114.
64. Kelley AS, Ettner SL, Morrison RS, Du Q, Wenger NS, Sarkisian CA. Determinants of medical expenditures in the last 6 months of life. Annals of internal medicine. 2011;154(4):235-42. Epub 2011/02/16. doi: 10.7326/0003-4819-154-4-201102150-00004. PubMed PMID: 21320939; PMCID: PMC4126809.
65. Leung JM, Udris EM, Uman J, Au DH. The effect of end-of-life discussions on perceived quality of care and health status among patients with COPD. Chest. 2012;142(1):128-33. Epub 2012/01/14. doi: 10.1378/chest.11-2222. PubMed PMID: 22241761; PMCID: Pmc3418855.
66. Abel J, Pring A, Rich A, Malik T, Verne J. The impact of advance care planning of place of death, a hospice retrospective cohort study. BMJ supportive & palliative care. 2013;3(2):168-73. Epub 2013/04/30. doi: 10.1136/bmjspcare-2012-000327. PubMed PMID: 23626905; PMCID: Pmc3632964.
67. Silveira MJ, Kim SY, Langa KM. Advance directives and outcomes of surrogate decision making before death. The New England journal of medicine. 2010;362(13):1211-8. Epub 2010/04/02. doi: 10.1056/NEJMsa0907901. PubMed PMID: 20357283; PMCID: Pmc2880881.
68. Berkman ND, Sheridan SL, Donahue KE, Halpern DJ, Viera A, Crotty K, Holland A, Brasure M, Lohr KN, Harden E, Tant E, Wallace I, Viswanathan M. Health literacy interventions and outcomes: an updated systematic review. Evidence report/technology assessment. 2011(199):1-941. Epub 2011/03/01. PubMed PMID: 23126607; PMCID: PMC4781058.
69. Waite KR, Federman AD, McCarthy DM, Sudore R, Curtis LM, Baker DW, Wilson EA, Hasnain-Wynia R, Wolf MS, Paasche-Orlow MK. Literacy and race as risk factors for low rates of advance directives in older adults. Journal of the American Geriatrics Society. 2013;61(3):403-6. Epub 2013/02/06. doi: 10.1111/jgs.12134. PubMed PMID: 23379361; PMCID: PMC3891653.
70. Tulsky JA, Fischer GS, Rose MR, Arnold RM. Opening the black box: how do physicians communicate about advance directives? Annals of internal medicine. 1998;129(6):441-9. Epub 1998/09/12. PubMed PMID: 9735081.
71. Volandes AE, Ferguson LA, Davis AD, Hull NC, Green MJ, Chang Y, Deep K, Paasche-Orlow MK. Assessing end-of-life preferences for advanced dementia in rural patients using an educational video: a randomized controlled trial. Journal of palliative medicine. 2011;14(2):169-77. Epub 2011/01/25. doi: 10.1089/jpm.2010.0299. PubMed PMID: 21254815.

72. Volandes AE, Barry MJ, Chang Y, Paasche-Orlow MK. Improving decision making at the end of life with video images. Medical decision making : an international journal of the Society for Medical Decision Making. 2010;30(1):29-34. Epub 2009/08/14. doi: 10.1177/0272989x09341587. PubMed PMID: 19675323.
73. Volandes AE, Ariza M, Abbo ED, Paasche-Orlow M. Overcoming educational barriers for advance care planning in Latinos with video images. Journal of palliative medicine. 2008;11(5):700-6. Epub 2008/07/01. doi: 10.1089/jpm.2007.0172. PubMed PMID: 18588401.
74. Volandes AE, Paasche-Orlow MK, Barry MJ, Gillick MR, Minaker KL, Chang Y, Cook EF, Abbo ED, El-Jawahri A, Mitchell SL. Video decision support tool for advance care planning in dementia: randomised controlled trial. BMJ (Clinical research ed). 2009;338:b2159. Epub 2009/05/30. doi: 10.1136/bmj.b2159. PubMed PMID: 19477893; PMCID: PMC2688013.
75. Volandes AE, Mitchell SL, Gillick MR, Chang Y, Paasche-Orlow MK. Using video images to improve the accuracy of surrogate decision-making: a randomized controlled trial. Journal of the American Medical Directors Association. 2009;10(8):575-80. Epub 2009/10/08. doi: 10.1016/j.jamda.2009.05.006. PubMed PMID: 19808156; PMCID: PMC2782701.
76. Volandes AE, Lehmann LS, Cook EF, Shaykevich S, Abbo ED, Gillick MR. Using video images of dementia in advance care planning. Archives of internal medicine. 2007;167(8):828-33. Epub 2007/04/25. doi: 10.1001/archinte.167.8.828. PubMed PMID: 17452547.
77. Epstein AS, Volandes AE, Chen LY, Gary KA, Li Y, Agre P, Levin TT, Reidy DL, Meng RD, Segal NH, Yu KH, Abou-Alfa GK, Janjigian YY, Kelsen DP, O'Reilly EM. A randomized controlled trial of a cardiopulmonary resuscitation video in advance care planning for progressive pancreas and hepatobiliary cancer patients. Journal of palliative medicine. 2013;16(6):623-31. Epub 2013/06/04. doi: 10.1089/jpm.2012.0524. PubMed PMID: 23725233.
78. Volandes AE, Levin TT, Slovin S, Carvajal RD, O'Reilly EM, Keohan ML, Theodoulou M, Dickler M, Gerecitano JF, Morris M, Epstein AS, Naka-Blackstone A, Walker-Corkery ES, Chang Y, Noy A. Augmenting advance care planning in poor prognosis cancer with a video decision aid: a preintervention-postintervention study. Cancer. 2012;118(17):4331-8. Epub 2012/01/19. doi: 10.1002/cncr.27423. PubMed PMID: 22252775; PMCID: PMC3359413.
79. Back AL, Arnold RM, Baile WF, Tulsky JA, Barley GE, Pea RD, Fryer-Edwards KA. Faculty development to change the paradigm of communication skills teaching in oncology. Journal of clinical oncology : official journal of the American Society of Clinical Oncology. 2009;27(7):1137-41. Epub 2009/01/28. doi: 10.1200/jco.2008.20.2408. PubMed PMID: 19171703; PMCID: PMC4804341.
80. Back AL, Arnold RM, Tulsky JA, Baile WF, Fryer-Edwards KA. Teaching communication skills to medical oncology fellows. Journal of clinical oncology :

official journal of the American Society of Clinical Oncology. 2003;21(12):2433-6. Epub 2003/06/14. doi: 10.1200/jco.2003.09.073. PubMed PMID: 12805343.
81. Clayton JM, Adler JL, O'Callaghan A, Martin P, Hynson J, Butow PN, Laidsaar-Powell RC, Arnold RM, Tulsky JA, Back AL. Intensive communication skills teaching for specialist training in palliative medicine: development and evaluation of an experiential workshop. Journal of palliative medicine. 2012;15(5):585-91. Epub 2012/03/22. doi: 10.1089/jpm.2011.0292. PubMed PMID: 22433021.
82. Clayton JM, Butow PN, Waters A, Laidsaar-Powell RC, O'Brien A, Boyle F, Back AL, Arnold RM, Tulsky JA, Tattersall MH. Evaluation of a novel individualised communication-skills training intervention to improve doctors' confidence and skills in end-of-life communication. Palliative medicine. 2013;27(3):236-43. Epub 2012/06/20. doi: 10.1177/0269216312449683. PubMed PMID: 22711714.
83. Fryer-Edwards K, Arnold RM, Baile W, Tulsky JA, Petracca F, Back A. Reflective teaching practices: an approach to teaching communication skills in a small-group setting. Academic medicine : journal of the Association of American Medical Colleges. 2006;81(7):638-44. Epub 2006/06/27. doi: 10.1097/01.acm.0000232414.43142.45. PubMed PMID: 16799286.
84. McGlynn EA, McClellan M. Strategies For Assessing Delivery System Innovations. Health affairs (Project Hope). 2017;36(3):408-16. Epub 2017/03/08. doi: 10.1377/hlthaff.2016.1373. PubMed PMID: 28264941.
85. Temel JS, Greer JA, Muzikansky A, Gallagher ER, Admane S, Jackson VA, Dahlin CM, Blinderman CD, Jacobsen J, Pirl WF, Billings JA, Lynch TJ. Early palliative care for patients with metastatic non-small-cell lung cancer. The New England journal of medicine. 2010;363(8):733-42. Epub 2010/09/08. doi: 10.1056/NEJMoa1000678. PubMed PMID: 20818875.
86. Hanson LC, Zimmerman S, Song MK, Lin FC, Rosemond C, Carey TS, Mitchell SL. Effect of the Goals of Care Intervention for Advanced Dementia: A Randomized Clinical Trial. JAMA internal medicine. 2016. Epub 2016/11/29. doi: 10.1001/jamainternmed.2016.7031. PubMed PMID: 27893884.
87. Bakitas M, Lyons KD, Hegel MT, Balan S, Brokaw FC, Seville J, Hull JG, Li Z, Tosteson TD, Byock IR, Ahles TA. Effects of a palliative care intervention on clinical outcomes in patients with advanced cancer: the Project ENABLE II randomized controlled trial. JAMA : the journal of the American Medical Association. 2009;302(7):741-9. Epub 2009/08/20. doi: 10.1001/jama.2009.1198. PubMed PMID: 19690306; PMCID: PMC3657724.
88. Bakitas MA, Tosteson TD, Li Z, Lyons KD, Hull JG, Li Z, Dionne-Odom JN, Frost J, Dragnev KH, Hegel MT, Azuero A, Ahles TA. Early Versus Delayed Initiation of Concurrent Palliative Oncology Care: Patient Outcomes in the ENABLE III Randomized Controlled Trial. Journal of clinical oncology : official journal of the American Society of Clinical Oncology. 2015;33(13):1438-45. Epub 2015/03/25. doi: 10.1200/jco.2014.58.6362. PubMed PMID: 25800768; PMCID: PMC4404422.
89. Thorpe KE, Zwarenstein M, Oxman AD, Treweek S, Furberg CD, Altman DG, Tunis S, Bergel E, Harvey I, Magid DJ, Chalkidou K. A pragmatic-explanatory

continuum indicator summary (PRECIS): a tool to help trial designers. Journal of clinical epidemiology. 2009;62(5):464-75. Epub 2009/04/08. doi: 10.1016/j.jclinepi.2008.12.011. PubMed PMID: 19348971.
90. Thorpe KE, Zwarenstein M, Oxman AD, Treweek S, Furberg CD, Altman DG, Tunis S, Bergel E, Harvey I, Magid DJ, Chalkidou K. A pragmatic-explanatory continuum indicator summary (PRECIS): a tool to help trial designers. CMAJ : Canadian Medical Association journal = journal de l'Association medicale canadienne. 2009;180(10):E47-57. Epub 2009/04/18. doi: 10.1503/cmaj.090523. PubMed PMID: 19372436; PMCID: PMC2679824.
91. Natural Language Processing to Assess End-of-Life Quality Indicators in Cancer Patients Receiving Palliative Surgery.
Lindvall C, Lilley EJ, Zupanc SN, Chien I, Udelsman BV, Walling A, Cooper Z, Tulsky JA.
J Palliat Med. 2019 Feb;22(2):183-187. doi: 10.1089/jpm.2018.0326. Epub 2018 Oct 17.
92. Needle in a Haystack: Natural Language Processing to Identify Serious Illness.
Udelsman B, Chien I, Ouchi K, Brizzi K, Tulsky JA, Lindvall C.
J Palliat Med. 2019 Feb;22(2):179-182. doi: 10.1089/jpm.2018.0294. Epub 2018 Sep 22.
93. Measuring Processes of Care in Palliative Surgery: A Novel Approach Using Natural Language Processing.
Lilley EJ, Lindvall **C**, Lillemoe KD, Tulsky JA, Wiener DC, Cooper Z.
Ann Surg. 2018 May;267(5):823-825. doi: 10.1097
94. Dyer N, Sorra JS, Smith SA, Cleary PD, Hays RD. Psychometric properties of the Consumer Assessment of Healthcare Providers and Systems (CAHPS(R)) Clinician and Group Adult Visit Survey. Medical care 2012;50 Suppl:S28-34 doi: 10.1097/MLR.0b013e31826cbc0d
95. S. M, P. RH, N. EM, K. CP. Modern psychometric methods for estimating physician performance on the Clinician and Group CAHPS survey. Health Serv Outcomes Res Method 2013;13:109-23
96. Holmes-Rovner M, Kroll J, Schmitt N, et al. Patient Satisfaction with Health Care Decisions:The Satisfaction with Decision Scale. 1996;16(1):58-64 doi: 10.1177/0272989x9601600114
97. Brehaut JC, O'Connor AM, Wood TJ, et al. Validation of a decision regret scale. Med Decis Making 2003;**23**(4):281-92 doi: 10.1177/0272989x03256005
98. Goel V, Sawka C, Thiel E, Gort E, O'Connor AM. Randomized trial of a patient decision aid for choice of surgical treatment for breast cancer. *Medical Decision Making* 2001; 21:1-6
99. Feldman-Stewart D, Brundage MD, Tishelman C. A conceptual framework for patient-professional communication: an application to the cancer context. Psycho-oncology. 2005;14(10):801-9; discussion 10-1. Epub 2005/10/04. doi: 10.1002/pon.950. PubMed PMID: 16200514.
100. Temel JS, Greer JA, El-Jawahri A, Pirl WF, Park ER, Jackson VA, Back AL, Kamdar M, Jacobsen J, Chittenden EH, Rinaldi SP, Gallagher ER, Eusebio JR, Li Z, Muzikansky A, Ryan DP. Effects of Early Integrated Palliative Care in Patients

With Lung and GI Cancer: A Randomized Clinical Trial. Journal of clinical oncology : official journal of the American Society of Clinical Oncology. 2016:Jco2016705046. Epub 2016/12/29. PubMed PMID: 28029308.
101. Committee on Improving the Quality of Cancer Care: Addressing the Challenges of an Aging P, Board on Health Care S, Institute of M. In: Levit L, Balogh E, Nass S, Ganz PA, editors. Delivering High-Quality Cancer Care: Charting a New Course for a System in Crisis. Washington (DC): National Academies Press (US) Copyright 2013 by the National Academy of Sciences. All rights reserved.; 2013.
102. Bernacki R, Hutchings M, Vick J, Smith G, Paladino J, Lipsitz S, Gawande AA, Block SD. Development of the Serious Illness Care Program: a randomised controlled trial of a palliative care communication intervention. BMJ open. 2015;5(10):e009032. Epub 2015/10/08. doi: 10.1136/bmjopen-2015-009032. PubMed PMID: 26443662; PMCID: Pmc4606432.
103. Sudore RL, Boscardin J, Feuz MA, McMahan RD, Katen MT, Barnes DE. Effect of the PREPARE Website vs an Easy-to-Read Advance Directive on Advance Care Planning Documentation and Engagement Among Veterans: A Randomized Clinical Trial. JAMA internal medicine. 2017. Epub 2017/05/19. doi: 10.1001/jamainternmed.2017.1607. PubMed PMID: 28520838.
104. El-Jawahri A, Paasche-Orlow MK, Matlock D, Stevenson LW, Lewis EF, Stewart G, Semigran M, Chang Y, Parks K, Walker-Corkery ES, Temel JS, Bohossian H, Ooi H, Mann E, Volandes AE. Randomized, Controlled Trial of an Advance Care Planning Video Decision Support Tool for Patients With Advanced Heart Failure. Circulation. 2016;134(1):52-60. Epub 2016/07/01. doi: 10.1161/circulationaha.116.021937. PubMed PMID: 27358437; PMCID: PMC4933326.
105. Block SD, Clark-Chiarelli N, Peters AS, Singer JD. Academia's chilly climate for primary care. JAMA : the journal of the American Medical Association. 1996;276(9):677-82. Epub 1996/09/04. PubMed PMID: 8769544.
106. Patton MQ. Qualitative Research & Evaluation Methods. 4th Ed. Sage Publications Inc., Thousand Oaks, CA: 2015.
107. Hui D, Bruera E. Integrating palliative care into the trajectory of cancer care. Nature reviews Clinical oncology. 2016;13(3):159-71. Epub 2015/11/26. doi: 10.1038/nrclinonc.2015.201. PubMed PMID: 26598947; PMCID: PMC4772864.
108. Lincoln Y, Guba E. Naturalistic Inquiry. Sage Publ.1985; 117.
109. Hripcsak G, Mirhaji P, Low AF, Malin BA. Preserving temporal relations in clinical data while maintaining privacy. Journal of the American Medical Informatics Association : JAMIA. 2016;23(6):1040-5. Epub 2016/03/26. doi: 10.1093/jamia/ocw001. PubMed PMID: 27013522; PMCID: PMC5070517.
110. Emanuel LL, Emanuel EJ, Stoeckle JD, Hummel LR, Barry MJ. Advance directives. Stability of patients' treatment choices. Archives of internal medicine. 1994;154(2):209-17. Epub 1994/01/24. PubMed PMID: 8285816.
111. Davies M, Fleiss JL. Measuring agreement for multinomial data. Biometrics. 1982;38(4):1047–1051.
