# Supplementary material for: An Intervention to Increase Advance Care Planning Among Older Adults With Advanced Cancer: A Randomized Clinical Trial
Source: JAMA Netw Open. 2025 May 9;8(5):e259150. doi: 10.1001/jamanetworkopen.2025.9150 (PMC12065034; doi:10.1001/jamanetworkopen.2025.9150)
Supplement: Supplement 2. — eFigure. Stepped-Wedge Cluster Randomization Design Scheme of 29 Clinics in Three Health Care Systems eTable. Natural Language Processing Keyword Library [file jamanetwopen-e259150-s002.pdf]

Supplementary Online Content

Volandes AE, Chang Y, Lakin JR, et al. Effective and aligned communication for older adults with advanced cancer: a randomized clinical trial. *JAMA Netw Open.* 2025;8(5):e259150. doi:10.1001/jamanetworkopen.2025.9150

eFigure. Stepped-Wedge Cluster Randomization Design Scheme of 29 Clinics in Three Health Care Systems
eTable. Natural Language Processing Keyword Library

This supplementary material has been provided by the authors to give readers additional information about their work.

eFigure. Stepped-Wedge Cluster Randomization Design Scheme of 29 Clinics in Three Health Care Systems

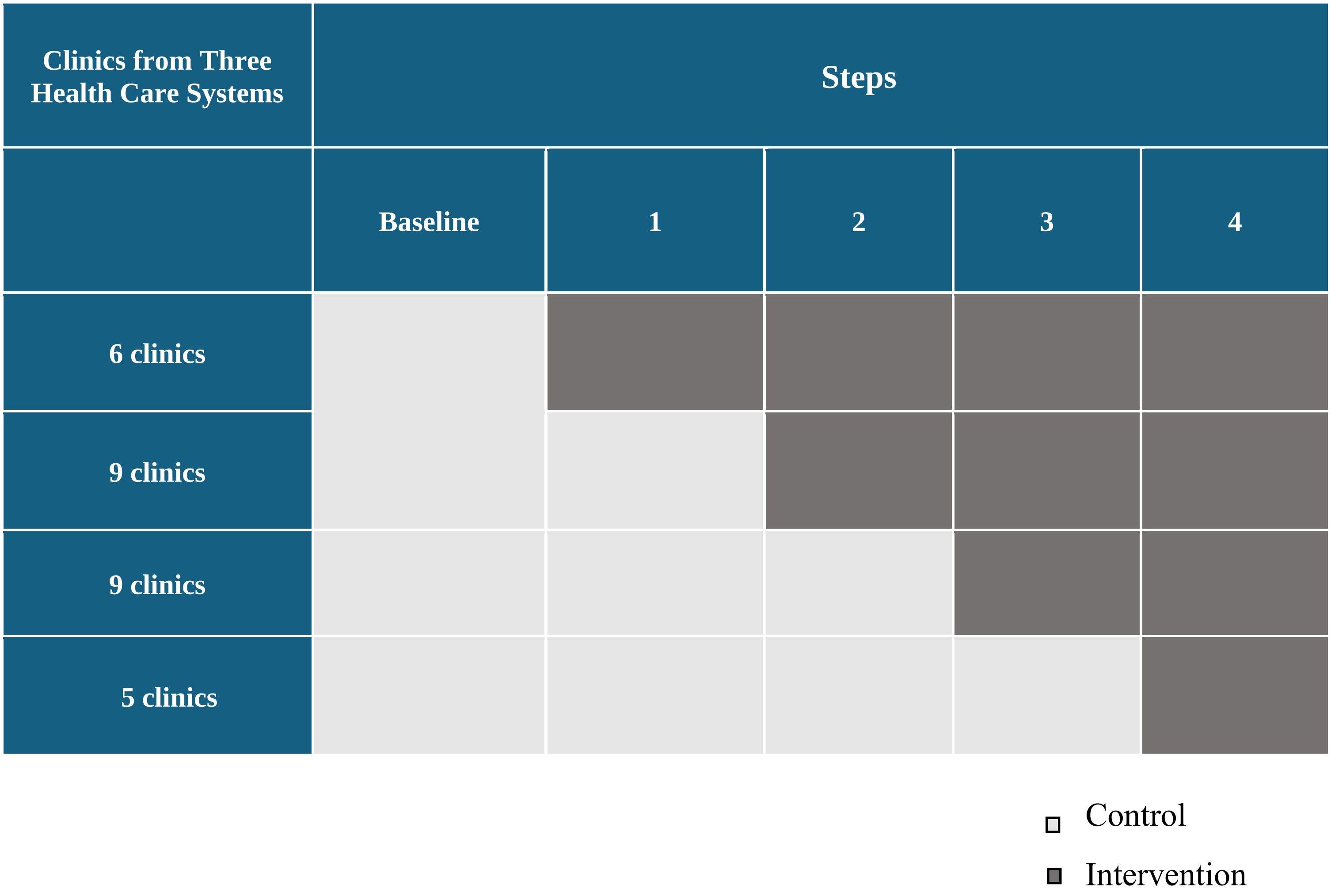

© 2025 Volandes AE et al. *JAMA Network Open.*

eTable. Natural Language Processing Keyword Library

Annotation Domain	Definition	Healthcare System A	Healthcare System B	Healthcare System C
Goals of Care	Conversations with patients or family members about the patient's goals, values, or priorities for treatment and outcomes. Includes statements that conversation occurred as well as listing specific goals.<br><br>OR<br><br>Advance care planning was discussed, reviewed, recommended, or completed.	goals for care, goals of treatment, goals for treatment, treatment goals, family meeting, family discussion, family discussions, patient goals, patient values, quality of life, prognostic discussions, illness understanding, serious illness conversation, serious illness discussion, acp, advance care planning, advanced care planning, comfort care, comfort approach, comfort directed care, advanced care plan/goals of care, comfort measures, end of life care, wish, NC Advanced Directive, living will, living wills, future goals, comfort	goc, goal of care, goals of care, goals for care, goals of treatment, goal of treatment, goals for treatment, treatment goals, treatment goal, family meeting, family discussion, family discussions, patient goals, patient values, quality of life, prognostic discussions, illness understanding, serious illness conversation, serious illness discussion, acp, advance care plan, advance care planning, advanced care planning, Supportive care, comfort care, comfort approach, comfort directed care, advanced care plan/goals of care, comfort measures, bend of life care, what matters most, wish, directive, directives,	goc, goals of care, goals for care, goals of treatment, goals for treatment, treatment goals, family meeting, family discussion, family discussions, patient goals, patient values, quality of life, prognostic discussions, illness understanding, serious illness conversation, serious illness discussion, acp, advance care plan, advance care planning, advanced care planning, Supportive care, comfort care, comfort approach, comfort directed care, advanced care plan/goals of care, comfort measures, end of life care, what matters most, wish

© 2025 Volandes AE et al. *JAMA Network Open.*

Palliative Care	Documentation that specialist palliative care was discussed, patient preferences regarding seeing palliative care clinician.	Palliative care, palliative medicine, pall care, pallcare, palcare, supportive care, comfort	palliative care, palliative medicine, pall care, pallcare, palcare,	Palliative care, palliative medicine, pall care, pallcare, palcare
Hospice	Documentation that hospice was discussed, prior enrollment in hospice, patient preferences regarding hospice, or assessments the patient did not meet hospice criteria.	Hospice	Hospice	Hospice
Code Status limitations	Conversations with patients or family members about preferences for limitations to cardiopulmonary resuscitation and intubation.	dnr, dnrdni, dni, dnr/dni, do not resuscitate, do-not-resuscitate, do not intubate, do-not-intubate, no intubation, no mechanical ventilation, no ventilation, no CPR, declines CPR, no cardiopulmonary resuscitation, chest compressions, no defibrillation, no dialysis, no NIPPV, no bipap, no endotracheal intubation, no mechanical intubation, declines dialysis, refuses dialysis, shocks, cmo, comfort measures, comfort care, Do not resuscitate/do not	dnr, dnrdni, dnr/dni, do not resuscitate, do-not-resuscitate, do not intubate, do-not-intubate, no intubation, no mechanical ventilation, no ventilation, no CPR, declines CPR, no cardiopulmonary resuscitation, chest compressions, no defibrillation, no dialysis, no NIPPV, no bipap, no endotracheal intubation, no mechanical intubation, declines dialysis, refuses dialysis, shocks,	dnr, dnrdni, dni, dnr/dni, do not resuscitate, do-not-resuscitate, do not intubate, do-not-intubate, no intubation, no mechanical ventilation, no ventilation, no CPR, declines CPR, no cardiopulmonary resuscitation, chest compressions, no defibrillation, no dialysis, no NIPPV, no bipap, no endotracheal intubation, no mechanical, intubation, declines dialysis, refuses dialysis, shocks, cmo, comfort measures, comfort care, Do not resuscitate/do not

© 2025 Volandes AE et al. *JAMA Network Open.*

		intubate, DNR/DNI/DNH, DNR/I, DNAR, heroic, comfort	cmo, comfort measures, comfort care, Do not resuscitate/do not intubate, dnr/dne/dnh, dnr/i, dnar,	intubate, DNR/DNI/DNH, DNR/I, DNAR, full code
Surrogate Decision Maker	Mention of a surrogate decision maker, including statements that a decision maker has been discussed or designated as well as naming or identification of a surrogate decision maker.	health care proxy, HCP, surrogate, decision maker, proxy, health agent, power of attorney for health care, HCPOA, health care power of attorney, health care agent, HCA, POA, Advanced Directives	health care agent, health care proxy, hcp, hcp agent, surrogate decision maker, proxy, health agent, power of attorney for health care, hcpoa, health care power of attorney, health care agent, hca, POA	health care agent, health care proxy, HCP, HCP agent, surrogate, decision maker, proxy, health agent, power of attorney for health care, HCPOA, health care power of attorney, health care agent, HCA
Video Decision Aid	Documentation that the ACP Decisions video decision support tool was recommended or vie	Video, decision aid, shared decision making, ACP decisions, ACP video	video, videos, decision aid, shared decision making, acp decisions,	video, videos, decision aid, shared decision making, ACP decisions

Example text: Correct usage

"During goals-of-care discussion with pt and family, pt expressed wish to be home"

Example text: Incorrect usage

"The patient indicated that walking with a cane instead of a walker is one of their goals of care."

© 2025 Volandes AE et al. *JAMA Network Open.*
