# Supplementary material for: An Intervention to Increase Advance Care Planning Among Older Adults With Advanced Cancer: A Randomized Clinical Trial
Source: JAMA Netw Open. 2025 May 9;8(5):e259150. doi: 10.1001/jamanetworkopen.2025.9150 (PMC12065034; doi:10.1001/jamanetworkopen.2025.9150)
Supplement: Supplement 3. — Data Sharing Statement [file jamanetwopen-e259150-s003.pdf]

Data Sharing Statement

Volandes. An Intervention to Increase Advance Care Planning Among Older Adults With Advanced Cancer. *JAMA Netw Open*. Published May 09, 2025.
doi:10.1001/jamanetworkopen.2025.9150

Data
Additional Information: Trial Registration Number NCT03609177
Data available: No

Additional Information
Explanation for why data not available: Individual health care systems will not share data.
